# Disulfiram attenuates cell and tissue damage and blood‒brain barrier dysfunction after intracranial haemorrhage by inhibiting the classical pyroptosis pathway

**DOI:** 10.1038/s41598-024-67118-2

**Published:** 2024-09-19

**Authors:** Chen Xu, Fangchao Jiang, Yuanfu Mao, Wan Wei, Jihe Song, Feihong Jia, Xinshu Du, Di Zhong, Guozhong Li

**Affiliations:** Department of Neurology, The First Affiliated Hospital of Harbin Medical University, Harbin, China

**Keywords:** Intracerebral haemorrhage, Disulfiram, Pyroptosis, GSDMD, Blood‒brain barrier, Stroke, Inflammasome, Blood-brain barrier, Cell death in the nervous system

## Abstract

No single treatment significantly reduces the mortality rate and improves neurological outcomes after intracerebral haemorrhage (ICH). New evidence suggests that pyroptosis-specific proteins are highly expressed in the perihaematomal tissues of patients with ICH and that the disulfiram (DSF) inhibits pyroptosis. An ICH model was established in C57BL/6 mice by intracranial injection of collagenase, after which DSF was used to treat the mice. Cell model of ICH was constructed, and DSF was used to treat the cells. HE, TUNEL, Nissl, FJC and IF staining were performed to evaluate the morphology of brain tissues; Western blotting and ELISA were performed to measure the protein expression of NOD-like receptor protein 3 (NLRP3)/Caspase-1/gasdermin D (GSDMD) classical pyroptosis pathway and Toll-likereceptor4 (TLR4)/nuclear factor-kappaB (NF-κB) inflammatory signaling pathway and blood‒brain barrier-associated factoes, and the wet/dry weight method was used to determine the brain water content. The expression of proteins related to the NLRP3/Caspase-1/GSDMD pathway and the TLR4/NF-κB pathway was upregulated in tissues surrounding the haematoma compared with that in control tissues; Moreover, the expression of the blood–brain barrier structural proteins occludin and zonula occludens-1 (ZO-1) was downregulated, and the expression of Aquaporin Protein-4 (AQP4) and matrix metalloprotein 9 (MMP-9) was upregulated. DSF significantly inhibited these changes, reduced the haematoma volume, decreased the brain water content, reduced neuronal death and degeneration and improved neurological function after ICH. ICH activated the classical pyroptosis pathway and TLR4/NF-κB inflammatory pathway, disruped the expression of blood–brain barrier structural proteins, and exacerbated brain injury and neurological dysfunction. DSF inhibited these changes and exerted the therapeutic effects on pathological changes and dysfunction caused by ICH.

## Introduction

Intracerebral haemorrhage (ICH) is a cerebrovascular disease that is characterized by haemorrhage within the brain parenchyma and has a mortality rate that can reach 50% within 30 days^1^. There is no single treatment that significantly improves neurological outcomes after ICH^2^.

During ICH, a lack of ATP and an increase in the concentrations of haematotoxic substances initiate a cascade of pathophusiological changes in pathophysiology, such as excitotoxicity, cellular oedema, the inflammatory response, oxidative stress, ionic disturbances and secondary disruption of the blood–brain barrier^3^. These pathophysiological changes may lead to various forms of cell death, including apoptosis, necrosis, necroptosis, autosis, and ferroptosis, which are all classified as types of programmed cell death^4^.

Pyroptosis, a mode of cell death that is heavily dependent on members of the gasdermin (GSDM) protein family, resulted in the formation of plasma membrane pores and was typically (but not always) the result of inflammatory caspase activation^5^. The classical pyroptosis, involves the activation of pro-caspase-1 via NLRP3 inflammasome activation, which is followed by the cleavage of GSDMD, pro-IL-1 and pro-IL-18 by activated caspase-1. Fragments of the GSDMD-N structural domain produced by cleavage are transported to the membrane, where they form circular pores in diameter. These pores are sufficient to allow the intracellular inflammatory factors to pass through, and these factors trigger cellular damage and inflammatory response. Our previous findings showed that pyroptosis-related proteins were expressed in the perihaematomal tissues of patients with ICH and that cells expressing high levels of these proteins exhibited characteristic structural changes that correlated the extent of which correlate with the onset time and severity of ICH^6^.

Disulfiram (DSF) as a drug originally used for treatment of alcoholism and has been shown to have oncostatic and anti-inflammatory effects in recent years^7^. In 2020, Hu et al.^8^ proposed that DSF inhibited cellular pyroptosis by inactivating active Cys via covalent modifications in a sepsis model.

The blood–brain barrier (BBB) is the modulating interface between the peripheral circulation and the central nervous system (CNS). Structural disruption or damage, can impair BBB function, resulting in cerebral oedema. Additionally, BBB disruption may further aggravate ionic homeostasis disruption, altered signaling, and immune infiltration, consequently causing cell death^3^. Several studies have attempted to investigate relationship between the expression of inflammasomes and upstream regulators of canonical inflammasomes and the BBB after ICH, by observing the neurobehavioral performance and brain water content^9^. However, few studies have explored the association between key protein, GSDMD, and BBB. The effect of pyroptosis on blood–brain barrier needs to be further verified.

In summary, the effects of pyroptosis on tissues after ICH and the mechanism have not been well studied^10^. The effects of pyroptosis on the BBB have mainly been studied in ischaemic stroke^11^ and have not been examined in the context of ICH. The ability of DSF to inhibit GSDMD and the related targets of DSF are still unknown^12^, and the effects of DSF on ICH have not yet been validated. Therefore, this study was designed to investigate the spatial and temporal patterns of pyroptosis and the damaging effects of pyroptosis on brain tissue and the BBB in mice subjected to ICH. We also assessed the effect of DSF on changes in the expression of genes and proteins as well as cellular, organizational, and behavioural changes after ICH to identify potential therapeutic targets.

## Materials and methods

### Animals

A total of 117 healthy male SPF-grade C57BL/6 mice (weighing approximately 22 g) were purchased from Liaoning Changsheng Biotechnology Co, Ltd, in China and housed in the Animal Centre of the First Affiliated Hospital of Harbin Medical University. All procedures involving animals treated strictly according to the Laboratory Animal Care guidelines from the First Afliated Hospital of Harbin Medical University, based on the recommendations of the US National Institutes of Health and the ARRIVE guidelines (https://arriveguidelines.org). And All procedures were approved by the Animal Ethics Committee of the First Hospital of Harbin Medical University (ethical lot number: 2021126). The mice were housed at room temperature (22 ± 1 °C) on a 12-h day/night cycle with a humidity of 60 ± 5% and were provided free access to water and food.

### Experimental design

#### Experiment 1 spatiotemporal expression patterns of GSDMD in perihaematomal tissues in mice subjected to ICH

Western blotting was used to measure GSDMD and IL-18 protein expression in perihaematomal tissues at 6 h, 12 h, 24 h, 72 h, and 7 d after ICH and in the sham operation group. Double immunofluorescence staining of GSDMD with ionized calcium binding adapter molecule 1 (IBA-1), glial fibrillary acidic protein (GFAP) and neuronal nuclear antigen (NeuN) was performed to assess the localization of GSDMD in perihaematomal tissues in the ICH and sham-operated groups at 72 h after ICH.

#### Experiment 2 effect of ICH on BV2 cell activity in mice and the therapeutic effect of DSF

The MTT assay was used to assess the effects of different concentrations of hemin (0, 5, 10, 20, 30 and 40 μM) on cellular activity to determine the IC50 of hemin; The effects of different concentrations of DSF (0, 0.25, 0.5, 1, 2, 4, 10, 20 and 40 μM) on cell activity were determined after treatment with the IC50 of hemin, and the EC50 concentration of DSF was used as the optimal therapeutic concentration.

#### Experiment 3 effects of DSF on short-term neurological deficits and haematoma expansion mice subjected to ICH

The effects of DSF at 3 doses of DSF (25 mg/kg, 50 mg/kg, 100 mg/kg) at 2 time points (24 h and 72 h after ICH) were assessed. Neurological function and the cerebral haematoma volume in the sham operation+vehicle group, ICH+vehicle group and ICH+DSF group were assessed in a blinded manner to determine the optimal therapeutic dose of DSF.

#### Experiment 4 effects of DSF on brain tissue morphology and microglia in ICH

The optimal therapeutic dose of DSF was administered to the animals at 1 time point (72 h after ICH). Brain tissue morphology in mice from the sham operation+vehicle group, ICH+vehicle group and ICH+DSF group was observed by HE, TUNEL, Nissl, FJC and IF staining.

The optimal therapeutic dose of DSF was administered to cells at 1 time point (24 h after ICH). Cell death and GSDMD expression in the control, ICH, ICH+DSF and ICH+vehicle groups were evaluated by TUNEL and double IF staining.

#### Experiment 5 in vivo and in vitro analysis of the effect of DSF on potential therapeutic targets

The protein expression of NLRP3, Caspase-1, ASC, GSDMD, IL-18, IL-1, TLR4, Myd88, PIRAK4, IRAK4, IRAK4, and p-NF-B and the gene expression of IL-18 and IL-1 in tissues around the haematoma in mice from the sham operation+vehicle group, the ICH+vehicle group, and the ICH+DSF group were assessed by Western blotting, qRT‒PCR and IF staining at 72 h after ICH.

The expression of NLRP3, Caspase-1, ASC, GSDMD, IL-18, IL-1 and NF-B in vitro in the control, ICH, ICH+DSF and ICH+vehicle groups was measured by Western blotting and ELISA.

#### Experiment 6 protective effect of DSF on the BBB in mice subjected to ICH

The dry and wet brain tissue weights in the sham operation+vehicle group, ICH+vehicle group and ICH+DSF group were recorded at 72 h after ICH. Western blotting was used to measure the expression of BBB-related proteins, and double IF staining was performed.

### Mouse models

#### ICH mouse model

The mice were randomly divided into five groups. In the ICH group, the mice were anaesthetized by an intraperitoneal injection of 0.3% pentobarbital sodium solution (65 mg/kg body weight). The bregma was used as the zero point, the microsyringe was placed 1.0 mm anterior and 2.2 mm to the left, and a hole was made in the cranium with a dental drill. A needle containing type VII collagenase solution (0.1 mg/ml, Sigma-Aldrich, China) was inserted vertically into the brain (to a depth of 3.2 mm from the skull surface), the needle was withdrawn 0.2 mm after being kept in place for 30 s, and 0.15 μL of the drug was injected into the brain tissue at a constant rate over a period of 10 min. Then, the needle was slowly removed after being kept in place for 5 min. The hole in the skull was then closed with bone wax. In the sham operation group, the mice were anaesthetized, craniotomy was performed, the microsyringe was inserted into the brain and withdrawn, and the hole in the skull was closed with bone wax. In the DSF+ICH group, 30 min after intragastric administration of DSF-DMSO-corn oil, ICH was induced as previously described. Then, DSF-DMSO-corn oil was intragastrically administered intragastrically twice per day at 8:00 a.m. and 8:00 p.m. Mice in the ICH+vehicle group were treated the same as those in the DSF+ICH group except that they were administered DMSO-corn oil rather than DSF-DMSO-corn oil. Mice in the sham operation+vehicle group underwent a sham operation and were intragastrically administered DMSO-corn oil. In the ICH group, body temperature was maintained at 37 °C, and the eyes were covered with wet gauze. There were four mice in each observation site subgroup. The mice were euthanized according to the experimental design for subsequent experiments. Any mice that died during ICH processing were excluded from the study.

#### Collection and processing of mouse brain tissue

After transcardial perfusion with precooled saline and 4% paraformaldehyde, the brain tissues were removed and fixed in 4% paraformaldehyde solution at 4 °C for 24 h. The tissues were subsequently dehydrated in 10–30% sucrose, embedded in OCT, snap frozen in liquid nitrogen, and sectioned at a thickness of 7 μm thick. Alternatively, the tissues were embedded in paraffin and sectioned at a thickness of 3 μm thick.

After transcardial perfusion with precooled saline, tissue around the haematoma was collected, and tissue homogenates were routinely prepared at 4 °C. The homogenates were lysed with lysis buffer (RIPA buffer, protease inhibitor, and phosphatase inhibitor), and the supernatant was collected by centrifugation for Western blot analysis. RNA was extracted from brain tissue with a kit according to the manufacturer’s instructions and analysed by qRT‒PCR.

### Culture of mouse BV2 cells

Resuspended BV2 cells were cultured in CM-0493 medium and in a carbon dioxide incubator (5% CO_2_, 37 °C). When the cell confluence reached approximately 85%, the cells were digested with trypsin, and the cells were passaged. According to the results of the cell viability and drug toxicity assays, the cells were cultured in blank medium overnight. BV2 cells were treated with hemin, DSF+hemin (DSF pretreatment for 30 min followed by the addition of hemin), or hemin+vehicle for 24 h, and the cells and supernatant were collected and saved for analysis.

### Determination of BV2 cell viability by the MTT assay

BV2 cells (5 × 10/100 μl) were inoculated into 96-well plates and treated with different concentrations of hemin (see Experiment 2) for 24 h, after which 10 μL of MTT solution was added, and the plates were incubated for 4 h. Then, 100 μl of methanol solution was added and the plates were incubated for 4 h. The absorbance was measured by an enzyme labelling instrument (at 570 nm), the cell viability was calculated (expressed as a percentage), and the optimal hemin concentration was determined. BV2 cells were treated with different concentrations of DSF (see Experiment 2) were used to treat BV2 cells in 96-well plates for 30 min, and the cells were incubated with the optimal concentration of hemin for 24 h. Subsequent analyses were carried out as described above to determine the optimal therapeutic concentration of DSF. As previously mentioned, IC50 and EC50 were calculated^13^.

### Western blotting

The BCA method was used to measure the protein concentration. Routine sampling, electrophoresis, and membrane transfer were carried out, and the membranes were blocked in 5% skim milk for 1 h at room temperature, incubated overnight at 4 °C in primary antibodies against NLRP3 (abcam, T55651, 1:1000); caspase-1 (proteintech, 22915-1-AP, 1:500); ASC (santa, Sc-514414, 1:500); GSDMD (abcam, ab219800, 1:1000); IL-18 (proteintech, 10663-1-AP, 1:5000); IL-1β (affinity, AF4006, 1:500); GAPDH (Proteintech, 60004, 1:50000); NF-κB (affinity, AF5006, 1:1000); TLR4 (proteintech, 66350-1-Ig, 1:1000); Myd88 (affinity, AF5195, 1:1000); PIRAK4 (affinity, DF7567, 1:1000); IRAK4 (affinity, DF2667, 1:1000); p-NF-κB (affinity, AF2006, 1:1000); MMP9 (proteintech, 10375-2-AP, 1:100); AQP4 (proteintch, 16473-1-AP, 1:100); occludin (proteintech, WL01996, 1:100); ZO-1 (proteintech, 21773-1-AP, 1:1000), washed 5 times with TBST, incubated for 45 min at room temperature in secondary antibody, and washed 6 times with TBST. After being incubated with ultrasensitive developing solution, the membranes were placed in a chemiluminescence imaging system and imaged. ImageJ software was used to calculate the grey values of the bands.

### Immunofluorescence staining

As previously mentioned, double fluorescence staining was performed^14^. Frozen sections were blocked, incubated with primary antibodies against GSDMD (Santa, sc-393656, 1:50); IL-18 (proteintech, 10663-1-AP, 1:50); IL-1β cleaved (affinity, #AF4006, 1:100); IBA1 (santa, Sc-32725, 1:50; abcam, ab178846, 1:500); Neun (PTM-bio, Ptm-5313, 1: 50; abcam, ab177487, 1:100); GFAP (wanlei, Wl0836, 1: 200; santa, Sc-33673, 1:50); MMP9 (proteintech, 10375-2-AP, 1:100); AQP4 (proteintch, 16473-1-AP, 1:100); occludin (proteintech, WL01996, 1:100); ZO-1 (proteintech, 21773-1-AP, 1:1000); CD31 (santa, sc-353622, 1:50) overnight at 4 °C, washed with PBS 3 times, incubated with secondary antibody for 3 h at room temperature, washed with PBS 3 times, and mounted with a solution containing DAPI. A fluorescence microscope was used to observe and photograph the slides, and ImageJ software was used to calculate the fluorescence intensity and cell number.

### TUNEL and FJC staining

Frozen tissue sections and cell specimens were subjected to immunofluorescence staining as described above through incubation of the secondary antibody for costaining. Then, staining was performed with TUNEL and FJC kits according to the manufacturer’s instructions, and the slices were mounted with a solution containing DAPI. Apoptotic cells and degenerated neuronal cells that fluoresced green were observed under a fluorescence microscope, and the fluorescence intensity and cell number were calculated by ImageJ software.

### HE and Nissl staining

Paraffin sections were routinely deparaffinized and dehydrated. For HE staining, the sections were incubated with haematoxylin for 5 min, and eosin for 2 min, and then mounted. For Nissl staining, the sections were incubated with 0.5% toluidine blue for 30 min at 37 °C and mounted. The sections were observed and photographed under a microscope^15^.

#### Analysis of mouse neurobehavioural functions

The neurological function of the mice at 24 h and 72 h after ICH was assessed with modified Garcia scores and the cornering test^16^. In the cornering test, the mice were placed in a 30° corner of a box and allowed to freely turn left or right to exit the corner. The cornering test was performed 50 times, and the results are expressed as the percentage of right turns.

#### Determination of cerebral haematoma volume and cerebral oedema volume

Mice were euthanized 72 h after ICH, brain tissue was quickly removed, the cerebellum was discarded, and the tissue was fixed in 4% paraformaldehyde solution at 4 °C for 24 h and then sliced continuously from the frontal pole on a precooled slice mould (2 mm thick). Four slices of the haemorrhage site were selected and photographed. The volume of intracranial haemorrhage was calculated by ImageJ software as follows: the volume of intracranial haemorrhage = the largest area of haematoma X the number of haemorrhagic layers X the thickness of each layer X 0.5. The wet weight of the brain tissue slices was obtained, the tissues were dried in an oven at 90 °C for 48 h, and the dry weight was determined. The water content of brain tissue was calculated as (wet weight–dry weight)/wet weight × 100%^16^.

#### qRT‒PCR

Total RNA was isolated from perihaematomal tissues 72 h after ICH using an RNA extraction kit according to the manufacturer's instructions. The sequences of the primers used are shown followed. The relative expression levels of the target genes were analysed by using the 2-ΔΔCt method, and GAPDH was used as an internal control.

Il-1b F: GCAACTGTTCCTGAACTCAACT, R: ATCTTTTGGGGTCCGTCAACT;

Il-18 F: GACAGCCTGTGTTCGAGGATATG, R:TGTTCTTACAGGAGAGGGTAGAC.

GAPDH F: AGGTCGGTGTGAACGGATTTG, R: TGTAGACCATGTAGTTGAGGTCA.

### ELISA

BV2 cell culture supernatant was centrifuged for 10 min at 22 °C (12,000 rpm), and the supernatant was collected. IL-1β and IL-18 concentrations were determined using ELISA kits according to the manufacturer's instructions.

### Statistical analysis

The results of the experiments are expressed as the mean ± standard deviation. A t test, r one-way ANOVA or a non-parametric test followed by Tukey's test and GraphPad 10.0 software were used to compare the groups using GraphPad 10.0 software. *p* < 0.05 was considered to indicate statistical significance.

## Results

### Experiment 1. Spatiotemporal expression of GSDMD in the perihaematomal tissues of mice after ICH

**Figure 1:**
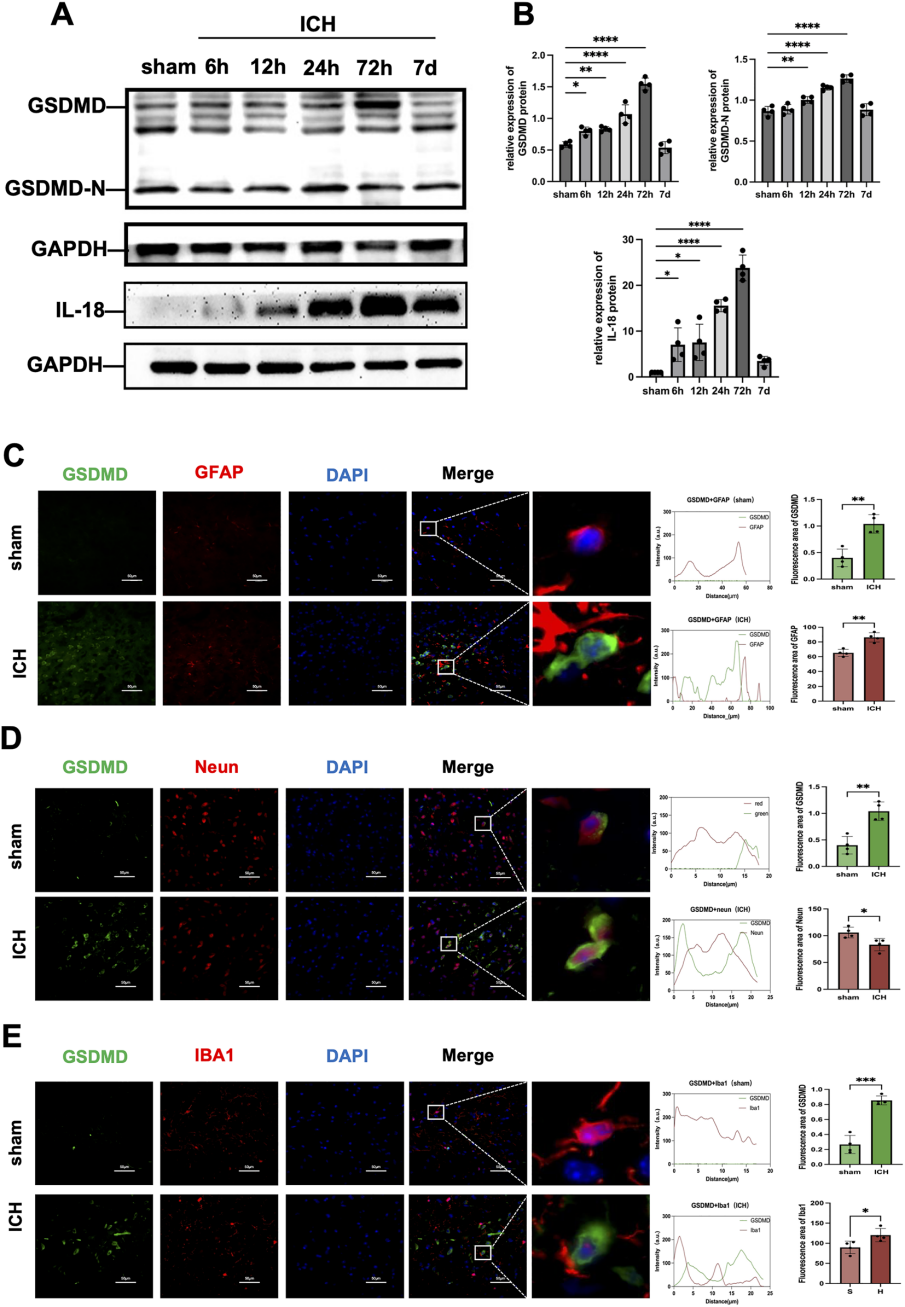
Spatiotemporal expression pattern of GSDMD in the perihaematomal tissues of mice subjected to ICH. (**A**) Relative protein levels of GSDMD and IL-18 in brain tissue at different time points after ICH were determined by Western blotting (n = 4). (**B**) The histogram shows the expression levels of GSDMD and IL-18 at different time points after ICH (n = 4). (**C**) Representative images of double-immunofluorescence staining showing the colocalization of DAPI (blue)/GSDMD (green)/GFAP (red). (**D**) Representative images of double-immunofluorescence staining showing the colocalization of DAPI (blue)/GSDMD (green)/Neun (red). (**E**) Representative images of double-immunofluorescence staining showing the colocalization of DAPI (blue)/GSDMD (green)/Iba1 (red) (n = 4). Scale bar = 100 µm. *p < 0.05, **p < 0.01, ***p < 0.001, ****p < 0.0001, N.S. not signifcant.

Western blot analysis revealed increased expression of GSDMD in the perihaematomal tissues of mice after ICH increased over time, and there was a significant increase in expression being observed at 24 h (*p* < 0.05). Moreover, GSDMD expression began to decrease after 72 h (Fig. 1A,B). Thus, 72 h after ICH was selected as the observation time point for subsequent animal experiments.

Double immunofluorescence staining revealed that GSDMD expression in the perihaematomal tissues of mice in the ICH group was significantly higher than that of mice in the sham-operated group at 72 h after ICH (*p* < 0.01) and that GSDMD was mainly localized to microglia and neuronal cells. Moreover, GSDMD expression was significantly increased in microglia (*p* < 0.01) and astrocytes and significantly decreased in neuronal cells (*p* < 0.05) (Fig. 1C–E). Thus, microglia were used for in vitro experiments, and neuronal damage was specifically studied in animal experiments.

### Experiment 2. Therapeutic effect of DSF on a BV2 cell model of ICH

**Figure 2:**
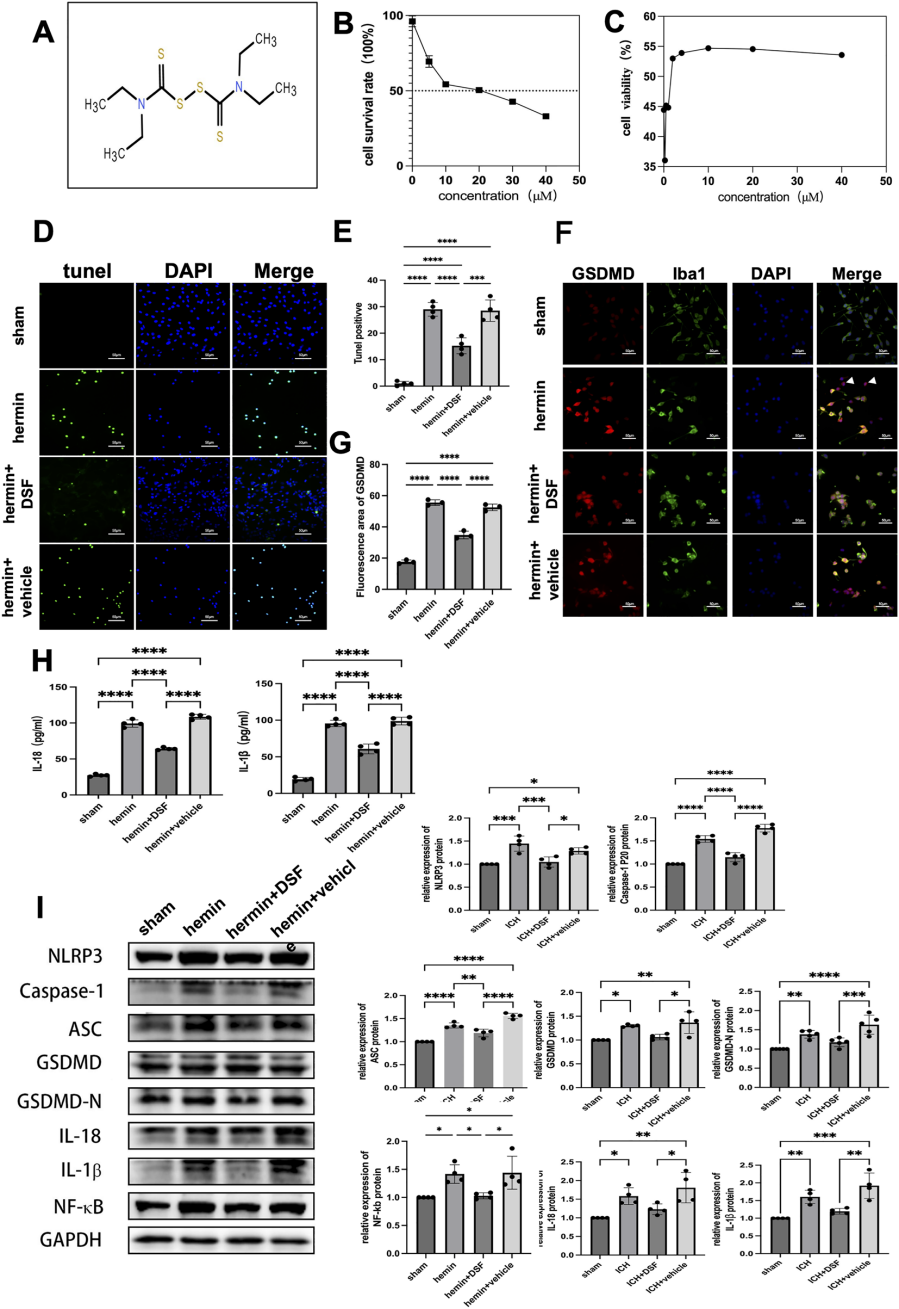
DSF attenuates hemin-induced BV2 cell damage by inhibiting cell death. (**A**) Molecular formula of disulfiram. (**B**, **C**) BV2 cell damage was induced by different concentrations of hemin, and the IC50 of hemin was determined by he MTT assay. Different concentrations of DSF inhibited hemin-induced cell damage, and the EC50 of DSF was determined by the MTT assay. (**D**, **E**) TUNEL assay showing cell death in the different groups. The results are presented as the means ± SDs. (**F**, **G**) Representative images of GSDMD/Iba1/nucleus (DAPI) triple-channel fluorescence staining in treated BV2 cells. Arrows indicate broken cells; scale bar = 50 μm. The results are presented as the means ± SDs. (**H**) The levels of IL-1β and IL-18 in the cell supernatants were determined by ELISA (n = 4). (**I**) Western blot analysis of the protein expression of NLRP3, Caspase-1, ASC, GSDMD, GSDMD-N, NF-κB, IL-18 and IL-1β protein in the different groups (n = 4). Representative Western blot images of NLRP3, caspase-1 P20, GSDMD, IL-1β, IL-18 and nf-kb and the internal reference control (GAPDH) in treated BV2 cells. (*p < 0.05, **p < 0.01, ***p < 0.001, **** *p* < 0.0001, ns. not significant).

The results of the MTT assay showed that the IC50 of hemin was 16.59 μM and the EC50 of DSF was 3.602 μM (Fig. 2B,C). Accordingly, in subsequent experiments, 15 μM hemin was chosen to induce brain cell death in mice, and 4 μM DSF was used as the therapeutic dose.

TUNEL staining confirmed an increase in the number of dead BV2 cells in the hemin group (Fig. 2D,E). Double immunofluorescence staining of GSDMD and Iba1 revealed that BV2 cells, BV2 cells in the hemin group were enlarged and rounded and had short protruding branches, and some of the cells exhibited staining that was inconsistent with nuclear staining (Fig. 2F). Analysis of the immunofluorescence intensity revealed a significant increase in GSDMD expression (Fig. 2G), and Western blotting revealed significantly increased expression of the pyroptosis-related proteins NLRP3, Caspase-1, ASC, GSDMD, GSDMD-N, NF-κB, IL-18 and IL-1β in the hemin group and the hemin+vehicle group (*p* < 0.05) (Fig. 2I). Compared with the hemin and hemin+vehicle groups, the DSF pretreatment+hemin group exhibited an increase in the number of BV2 cell nuclei (GSDMD/Iba1 colocalization), a significant reduction in cell death (TUNEL staining)(*p* < 0.0001) (Fig. 2D,E), and significantly reduced expression of GSDMD (immunofluorescence staining) (Fig. 2F,G), IL-1β and IL-18 (ELISA) (*p* < 0.0001) (Fig. 2H) as well as the pyroptosis-related proteins NLRP3, Caspase-1, ASC, NF-κB, IL-18, and IL-1β (Western blot analysis) (*p* < 0.05). Western blot analysis also revealed that the DSF pretreatment+hemin group exhibited significantly lower expression of the pyroptosis-related proteins GSDMD, GSDMD-N, IL-18 and IL-1β than the vehicle group (*p* < 0.05) (Fig. 2I).

### Experiment 3. DSF attenuates short-term neurological deficits and the haematoma volume in mice subjected to ICH

**Figure 3:**
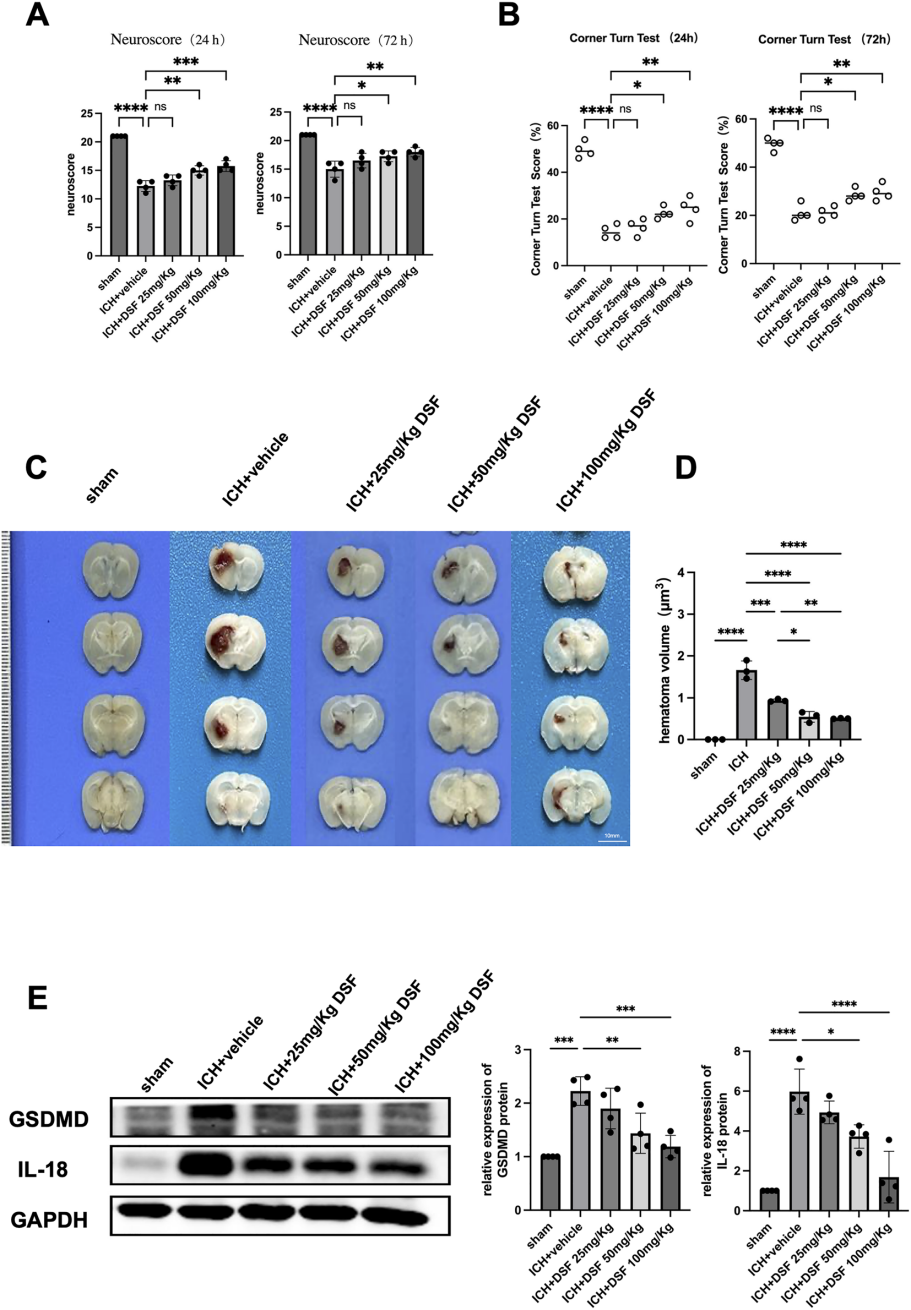
(**A**) Modified Garcia test scores at 24 h and 72 h (n = 4). (**B**) Corner turn test scores at 24 h and 72 h (n = 4). (**C**, **D**) Representative images and statistical analysis of the haematoma area after treatment with different doses of disulfiram (n = 3, scale bar = 5 mm). (**G**) Western blot showing the protein expression of GSDMD and IL-18 after treatment with different doses of disulfiram. (n = 4, *p < 0.05, **p < 0.01, ***p < 0.001, ****p < 0.0001; ns. not significant).

Mice in the ICH+vehicle group had significantly lower neuroscores and CTT scores than those in the sham-operated group at both 24 h and 72 h after ICH (*p* < 0.0001). Mice treated with 50 mg/kg DSF or 100 mg/kg DSF had significantly higher neuroscores and CTT scores than those treated with vehicle at 24 h and after ICH (*p* < 0.05) (Fig. 3A,B); The cerebral haematoma area of mice in the DSF+ICH group were significantly smaller than those of mice in the ICH+vehicle group (*p* < 0.01), and there were no significant difference in the haematoma areas between the 50 mg/kg DSF group and 100 mg/kg DSF group (Fig. 3C,D). The mortality rate of mice in the 100 mg/kg DSF group was higher than that of mice in the 50 mg/kg DSF group, and these findings were further supported by the Western blot results (Fig. 3E). Accordingly, 50 mg/kg DSF was administered by intraperitoneal injection 0.5 h after ICH in subsequent animal experiments.

### Experiment 4. DSF attenuates microstructural damage to brain tissue in mice subjected to ICH

**Figure 4:**
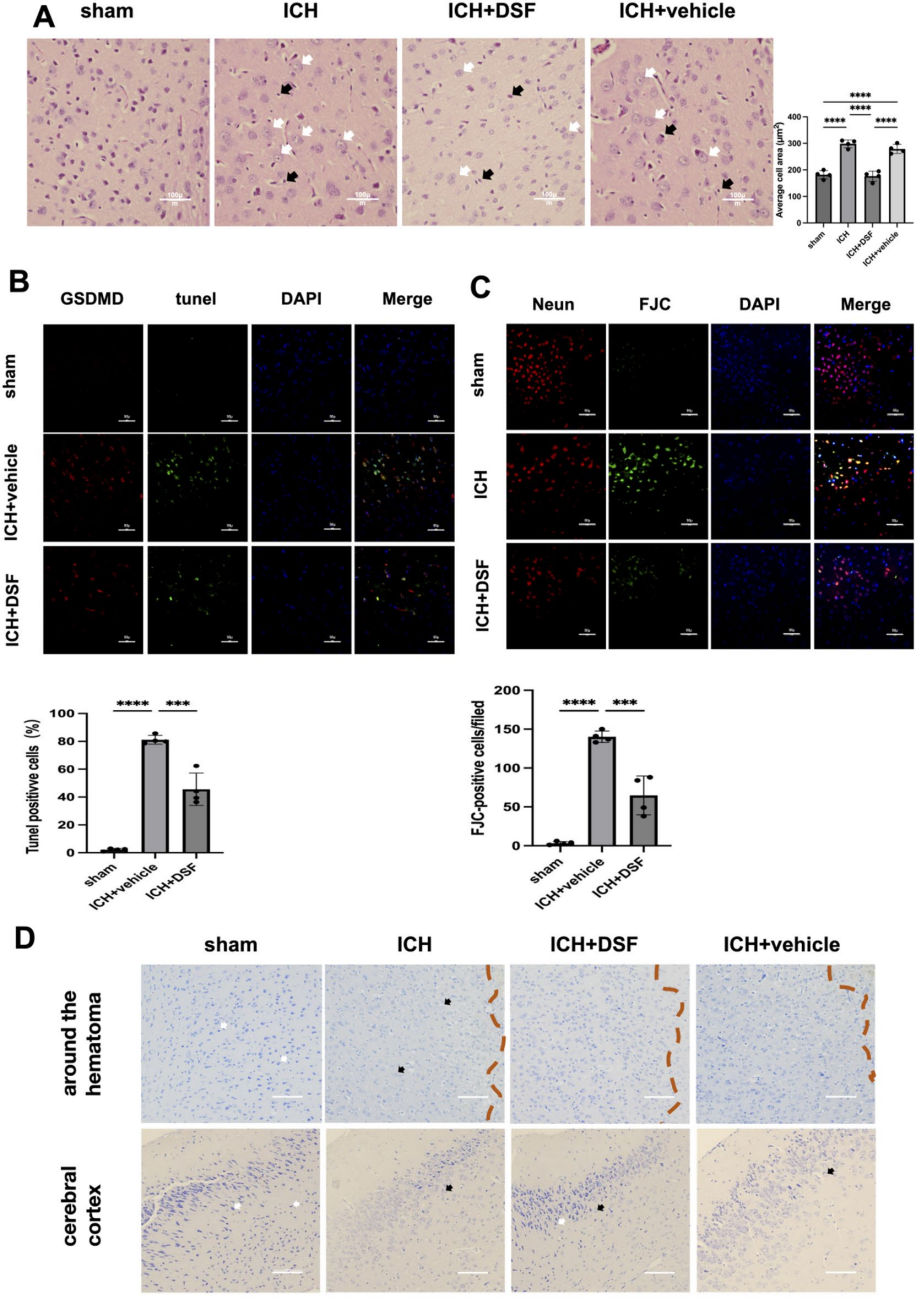
(**A**) H&E staining of the brain tissue of mice with brain haemorrhage caused by collagenase injection after the administration of disulfiram. The statistical chart shows the average cell area of each group. Vacuolated neuropils in brain tissue. White arrows point to distended cells with deeply stained/fragmented/dissolved nuclei, and black arrows point to neuronal nuclei, which were shrunken and trichromatic (n = 4. Scale bar = 50 μm). (**B**) TUNEL staining showing cell death in the different groups. The results are presented as the means ± SDs. (**C**) DAPI (blue)/FJC (green)/NeuN (red) representative immunofluorescence images. Immunofluorescence staining of the FJC (n = 4. Scale bar = 100 µm). (**D**) Nissl staining of the brain tissue of mice subjected to ICH after the administration of DSF. The white arrow points to neurons with Nissl bodies. The black arrow points to neurons lacking Nissl bodies (n = 4). Scale bar = 50 µm. *p < 0.05, **p < 0.01, ***p < 0.001, ****p < 0.0001, N.S. not significant. The data are presented as the mean ± SD.

HE staining revealed that the brain tissue in the sham-operated group was clear and intact, with neatly arranged nerve cells and no oedema. The tissue around the haematoma in the ICH group and the ICH+vehicle group, had swollen, lacked myelin sheaths and had sparse nerve fibres. In these groups, the nuclei of peripheral neurons were pyknotic and darkly stained, glial cells were increased in number and exhibited swollen cytosols, the number of dark-stained/fragmented/dissolved nuclei was significantly increased, and the number of hollowed and vacuolated cells was significantly increased. Compared with the ICH group, the DSF+ICH group showed reduced number of pyknotic nuclei with darker staining and an indistinct structure, significantly reduced cytosolic swelling in glial cells, reduced dark staining/fragmentation/dissolution of nuclei, and reduced number of hollowed and vacuolated cells (Fig. 4A).

TUNEL staining revealed that there were many dead cells in the tissue surrounding the haematoma in the ICH+vehicle group. Specifically, there were significantly more dead cells in the ICH+vehicle group than in the sham operation group (*p* < 0.0001). Moreover, there were significantly fewer dead cells in the DSF+ICH group than in the ICH+vehicle group (*p* < 0.001) (Fig. 4B).

FJC staining revealed significantly more FJC-positive cells in the ICH+vehicle group (*p* < 0.0001) and substantially fewer FJC-positive cells in the DSF+ICH group than in the sham operation group (*p* < 0.001) (Fig. 4C).

Nissl staining was performed, and Nissl bodies were clearly visible and evenly distributed in brain tissue in the sham-operated group. In the ICH group and the ICH+vehicle group, the number of Nissl bodies in the tissue surrounding the haematoma and cerebral cortex was significantly reduced, and damaged cells with pyknotic and dark-stained nuclei and swollen cytosols were observed. Compared with the sham-operated group, the DSF+ICH group had fewer Nissl bodies in the tissues surrounding the haematoma, but the degree to which the number of Nissl bodies was reduced was significantly lower in the DSF+ICH group than in the ICH group and the ICH+vehicle group. Numerous Nissl bodies were observed in neuronal cells, and the degree of damage and cell swelling was reduced (Fig. 4D).

### Experiment 5. DSF inhibits the pyroptosis pathway and TLR4/NF-κB pathway in mice subjected to ICH

**Figure 5:**
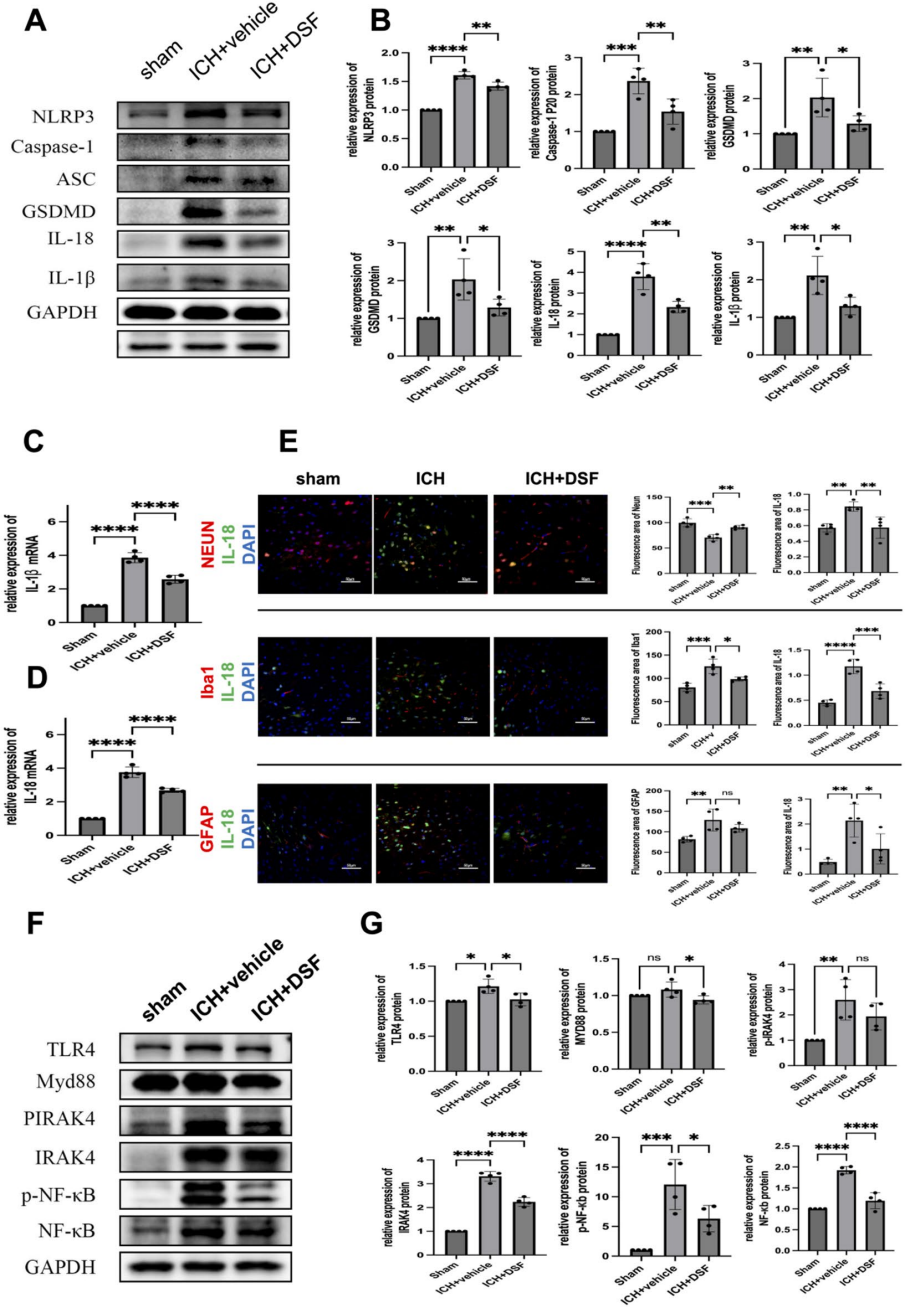
(**A**, **B**) Western blot showing the protein expression of NLRP3, caspase-1, ASC, GSDMD, IL-1β, and IL-18 in the different groups (n = 4). (**C**, **D**) qRT‒PCR results showing the levels of IL-1beta and IL-18 in the different groups (n = 4). (**E**) IL-18 colocalization with IBA1, Neun, and GFAP in the sham group, model+solvent group, and treatment group, and the results were statistically analysed. (**F, G**) Western blot showing the protein expression of TLR4, Myd88, Pirak4, irak4, p-nf-kb, and nf-kb in the different groups. The results are presented as the means ± SDs. (n = 4, *p < 0.05, **p < 0.01, ***p < 0.001, **** *p* < 0.0001; ns. not significant).

Western blot analysis revealed that the expression of the pyroptosis-related proteins NLRP3, Caspase-1, ASC, GSDMD, IL-18 and IL-1β in the tissues surrounding the haematoma was significantly higher in the ICH group than in the sham-operated group (*p* < 0.01) and lower in the DSF+ICH group than in the ICH+vehicle group (*p* < 0.05) (Fig. 5A).

qRT‒PCR showed that the expression of the pyroptosis-related genes IL-1β and IL-18 in the tissues surrounding the haematoma was significantly higher in the ICH group than in the sham-operated group (*p* < 0.0001) and significantly lower in the DSF+ICH group than in the ICH group and the ICH+vehicle group (*p* < 0.0001) (Fig. 5C,D).

Triple immunofluorescence staining revealed that the protein expression of IL-18 protein in the tissue surrounding the haematoma was increased, the localization of IL-18 whit microglia, astrocytes and neuronal cells was increased, and the number of glial cells and glial cell activation were increased in the ICH group compared with the sham-operated group. Moreover, the protein expression of IL-18 protein in the tissue surrounding the haematoma and the severity of microglial and neuronal cell damage in the DSF+ICH group were significantly lower than those in the ICH+vehicle group (*p* < 0.05) (Fig. 5E).

The expression of the TLR4/NF-B pathway-related proteins TLR4, PIRAK4, IRAK4, NF-κB and p-NF-κB in perihaematomal tissues was significantly higher in the ICH group than in the sham-operated group (*p* < 0.05);. Moreover, the protein expression of Myd88 was increased in the ICH group compared with the sham-operated group, but the difference was not statistically significant. Except PIRAK4 expression, these parameters except PIRAK4 expression were lower in the DSF+ICH group than in the ICH+vehicle group (*p* < 0.05) (Fig. 5F,G).

### Experiment 6. DSF protects against BBB injury in mice subjected to ICH

**Figure 6:**
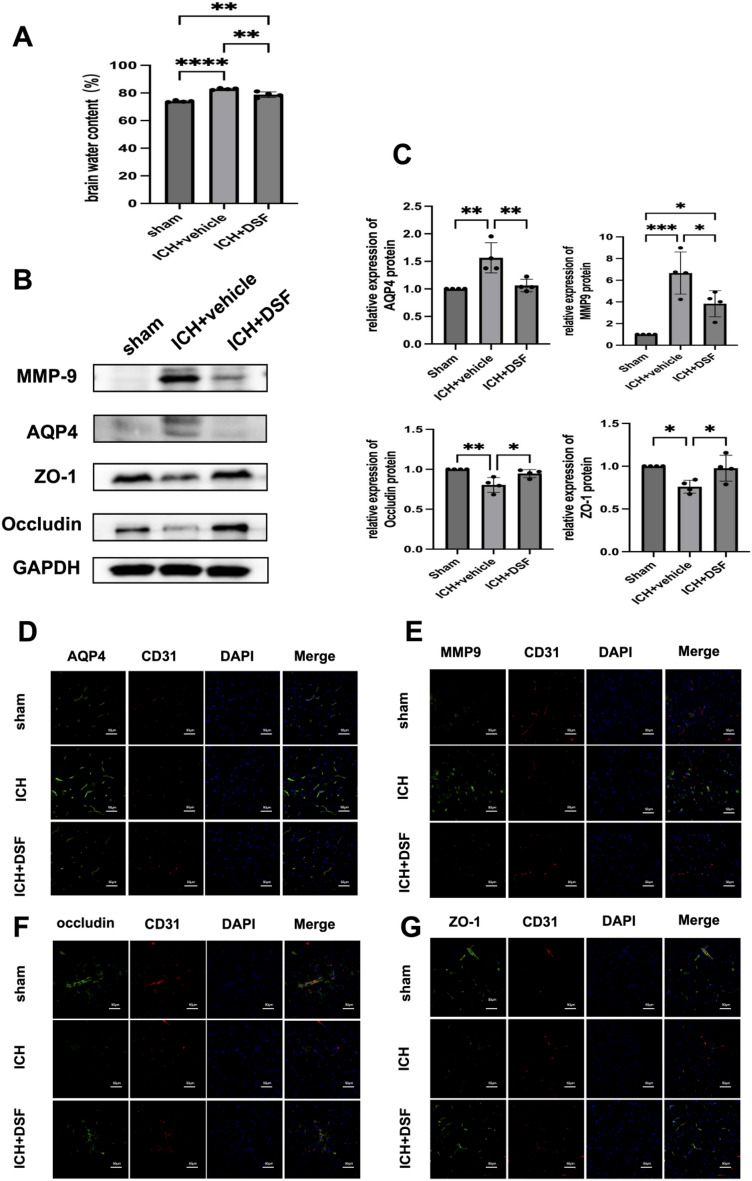
DSF protects against BBB injury in mice subjected to ICH. (**A**) Brain water content (n = 4). (**B**, **C**) Western blot showing the protein expression of AQP4, MMP9, occludin and ZO-1 protein in the different groups (n = 4). (**D**) Representative images of double-immunofluorescence staining showing the colocalization of DAPI (blue)/AQP4 (green)/CD31 (red), (n = 4). Scale bar = 100 µm. (**E**) Representative images of double-immunofluorescence staining showing the colocalization of DAPI (blue)/MMP-9(green)/CD31 (red), (n = 4). Scale bar = 100 µm. (**F**) Representative images of double-immunofluorescence staining showing the colocalization of DAPI (blue)/occludin (green)/CD31 (red), (n = 4). Scale bar = 100 µm. (**G**) Representative images of double-immunofluorescence staining showing the colocalization of DAPI (blue)/ZO-1 (green)/CD31 (red) (n = 4). Scale bar = 100 µm,*p < 0.05, **p < 0.01, ***p < 0.001, ****p < 0.0001, ns. not significant.

The brain water content was significantly higher in the ICH group than in the sham operation group (*p* < 0.0001), and was significantly lower in the DSF+ICH group than in the ICH group (*p* < 0.01) (Fig. 6A).

Western blot analysis revealed that the protein expression of ZO-1 and occludin was significantly decreased and the protein expression of AQP4 and MMP-9 was significantly increased in the ICH group compared with the sham-operated group (*p* < 0.05); Moreover, the protein expression of ZO-1 and occludin was significantly higher and the protein expression of AQP4 and MMP-9 was significantly lower in the DSF+ICH group than in the ICH+vehicle group (*p* < 0.05) (Fig. 6B,C).

Double immunofluorescence staining revealed that the colocalization of occludin and ZO-1 with the vascular endothelial marker CD31, in perihaematomal tissues was significantly reduced in the ICH group compared with the control group, Moreover, the distribution of AQP4 in the perivascular area was increased in the ICH group, and AQP4 staining was detected in extravascular areas. The expression of occludin and ZO-1 and the colocalization of occludin and ZO-1 with CD31 were significantly increased in the DSF+ICH group, and the immunofluorescence intensity of perivascular AQP4 was reduced. In contrast, the opposite changes occurred in MMP9 and occludin (Fig. 6D–G).

## Discussion

Several studies in recent years have shown that pyroptosis plays a role in brain tissue injury after ICH. Inhibiting pyroptosis-mediated neuroinflammation and tissue factor release can alleviate neurological dysfunction after ICH^17,18^.

In this study, we established ICH models in mice and BV2 cells. Western blot analysis and double immunofluorescence stainingwas used to evaluate the spatiotemporal and temporal patterns of GSDMD expression in the tissues surrounding the haematoma. The results showed that the expression of GSDMD increased over time and peaked at 72 h. GSDMD was mainly localized to microglia and neuronal cells. Moreover, microglial GSDMD expression in experiments demonstrated that the expression of relevant proteins and genes associated with the NLRP3/Caspase-1/GSDMD pathway was upregulated in perihaematomal tissues and in an ICH model in BV2 cells and their supernatants, suggesting that the classic pyroptosis pathway was activated in mice subjected to ICH.

Consistent with our results, several animal studies have confirmed an increase in GSDMD levels is after ICH. Inflammasomes are activated after ICH through multiple pathways, including the classic NLRP3/caspase-1 pathway, oxidative damage/NLRP3/caspase-1 pathway, tumour necrosis factor receptor-associated factor 6 (TRAF6)/NLRP3 pathway, dopamine D1 receptor (DRD1)/NLRP3/caspase-1 pathway and nuclear factor erythroid-2 related factor 2 (Nrf2)/NLRP3/caspase-1 pathway. Moreover, erythrocyte lysis and endoplasmic reticulum (ER) stress can activate NLRP3, and the levels of proteins associated with NLRP3/caspase-1/GSDMD increase^10^. GSDMD can also be increased through the ASC/Caspase-1/GSDMD pathway^9^, the NK1R/PKCδ pathway^19^, and the PI3K/AKT pathway^20^.

In other disease models, an increase in GSDMD may be associated with the TLR4/NF-κB pathway^21^.

DSF, which is an acetaldehyde dehydrogenase inhibitor, is a FDA-approved drug for the treatment of alcoholism and has been in clinical use for more than 60 years. Recent studies have suggested that DSF also has antitumour^22^, anti-inflammatory^23^, ant pyroptotic effects^8^. Since this compound shows good bioavailability and can cross the BBB^24^, it has the potential to become a therapeutic agent for diseases associated with nerve damage and inflammation. However, research on DSF has focused mainly on nonneurological injury and inflammatory diseases, although its efficacy in the treatment of gliomas^25^, spinal cord injuries and ischaemic stroke^26^ has been studied. Our previous study showed increases in NLRP3, Caspase-1, and GSDMD expression and the number of cells showing typical characteristics of pyroptosis in tissues surrounding cerebral haematomas in humans.

To determine whether DSF can inhibit the release of inflammatory factors mediated by pyroptosis and thereby alleviate neurological dysfunction after ICH, we pretreated mice with DSF after ICH and treated an ICH model in BV2 cells with DSF. Histomorphological analysis revealed that DSF treatment effectively reduced the haematoma area, alleviated oedema, reduced glial cell activation and damage and neuronal cell death and degeneration, and alleviated behavioural deficits. In vitro and in vivo studies revealed that DSF treatment reduced the expression of proteins related to the classical NLRP3/Caspase-1/GSDMD pyroptosis pathway and the TLR4/NF-κB inflammatory signalling pathway after ICH.

Based on these results, we suggest that DSF alleviates early brain injury after ICH by inhibiting the classical NLRP3/Caspase-1/GSDMD pyroptosis pathway, reducing the ability of the N-terminus of GSDMD to form pores in the cellular membrane, inhibiting the activation of the NLRP3 inflammasome activation and inducing the release of inflammatory factors into the mesenchyme to further induce an inflammatory cascade. The findings of this study on the inhibitory effects of GSDMD and IL-1β expression are consistent with those of Zhao et al.^27^. The possible reason is that DSF, In addition to having a perforation inhibitory effect against the GSDMD N-terminus, DSF also reduces the expression of proteins associated with the TLR4 inflammatory pathway, which decreases the expression of downstream inflammatory factors. The difference in Hu's findings may be due to differences in disease models and experimental subjects.

Whether DSF has a protective or destructive effect on cells remains controversial. The results of our study showed that DSF exerted a protective effect on cells, which was consistent with the results of several previous studies^28,29^. Other studies came to the opposite conclusion, however, and suggested that DSF exerted inhibitory and destructive effects on cancer cells such as glioma cells^30^. A literature review indicated that the pharmacological mechanisms of action of DSF are complex and diverse and require further in-depth study^31^.

The protective effect of DSF on tissues and cells is due to its inhibitory effect on pyroptosis. An earlier study, involving high-throughput screening and subsequent in vitro and in vivo experiments, revealed that treatment with DSF inactivated active Cys residues through covalent modifications in a sepsis model^32^. In a study of an LPS-induced peritonitis model and an MSU-induced gout model^33^, Deng et al. reported that DSF prevented the release of lysosomal protease B from lysosomes into the cytoplasm and directly inactivated the NLRP3 inflammasome, thereby inhibiting the activation of downstream pyroptosis pathways^23^. NLRP3 can be activated by NF-κB, and DSF not only inhibits NF-κB but also reduces the production of NF-κB by blocking the TLR4 signalling pathway, which reduces the activation of NLRP3 by NF-κB, thereby suppressing pyroptosis^12^. DSF may exert many effects on pyroptosis, and there is no standardized method for researching these effects, which may be the reason for the contradictory experimental findings.

In our study, DSF reduced neuronal cell destruction and death, possibly by inhibiting the TLR4/NF-κB pathway and NLRP3 activation, as well as through the cleavage of GSDMD, which reduced pore formation in the cell membrane and cell rupture caused by pyroptosis, thereby reducing the levels of proinflammatory cytokines released to the interstitium and inhibiting the inflammatory response.

In this study, we first verified that the administration of DSF after ICH could alleviate brain oedema and BBB damage and then analysed the possible underlying mechanisms.

DSF increased the protein expression of occludin and ZO-1 in the tissues surrounding the haematoma after ICH. Major tight junction proteins include claudin-5, occludin and JAM-A, which interact with proteins on neighbouring cells to help close the paracellular space. The tight junction protein ZO-1 is involved in forming the cytoskeleton. Administration of DSF reduced the protein expression of MMP-9 in the tissue surrounding the haematoma after ICH. It has been shown that matrix metalloproteinase (MMP) levels are significantly increased after ICH, leading to BBB breakdown through the direct degradation of connexins, and that claudin-5 and occludin are substrates of MMPs^34^. In a mouse model of middle cerebral artery occlusion, inhibition of MMP-9 expression resulted in increased ZO-1 and occludin expression and reduced BBB permeability^35^. An increase in MMP-9 expression is associated with haematoma expansion, oedema, and neurological deterioration in patients.

As previously described, DSF administration reduced the expression of proteins related to the classical pyroptosis pathway and the TLR4/NF-κB inflammatory signalling pathway in the tissues surrounding the haematoma after ICH, thereby inhibiting the subsequent inflammatory response. Cytokines are the main mediators of the inflammatory response and are important factors in the destruction of the BBB, which leads to brain oedema^36^. Caspase-1 inhibitors have been shown to protect the BBB during the acute phase of stroke^11^.

When brain-resident microglia are activated during the early stages of ICH, proinflammatory cytokines are released^37^. IL-1β is typically present at low or undetectable levels in the brain and can be rapidly upregulated in models of various experimental brain injury. IL-1β precursors must be cleaved and processed by caspase-1 to produce active IL-1β, which enters the interstitium through disrupted cell membranes, where it exerts its effects^36^. Activation of the TLR4/NF-κB signalling pathway results in the production of additional proinflammatory cytokines, including TNF-α. Li et al. demonstrated that the administration of an NF-κB inhibitor to reduce the nuclear translocation of NF-κB not only suppressed TNF-α production in microglia but also inhibited EC necrosis in a coculture system. NF-κB inhibitors reduce cerebral oedema^38^.

In conclusion, this study demonstrated for the first time that DSF could protect the BBB after cerebral haemorrhage by inhibiting specific pathways and targets reducing the level of peripheral oedema and alleviating related neurological dysfunction. Thus, this study verified the protective effect of DSF on the BBB in an ICH model.

Importantly, the optimal dose of DSF for treating ICH in vivo was also determined in this study; Previous studies and the preliminary conclusions of the present study suggest that the therapeutic effect of DSF on cell death may be dose-dependent, i.e., DSF may have a therapeutic effect at a low dose but may be neurotoxic at a high dose^39^. Further examiation of the dose-dependent effects of DSF is needed to validate this hypothesis.

The present study was also limited by the fact that a single time point was chosen for observation. A comparison of the treatment effects of DSF at multiple time points would be a better way to study its early and long-term effects. The effect of DSF on the BBB should also be examined early in the disease to allow for more robust conclusions to be drawn.

In the present study, male animals were selected for the study, and experiments were not performed all sexes or in animals with various health statuses, which may introduce bias into the experimental conclusions. Future relevant studies will include experimental subjects of different sexes and different health states to examine the clinical applicability of this drug^40^.

In this study, only BV2 cells were used in vitro, and only in vivo experiments were carried out to examine the protective effects on neurons. In addition, we did not investigate pyroptosis or the effects of drugs on blood–brain barrier cells, and our research team will further explore the effects of DSF on various cells after ICH and the related mechanisms (Fig. S1).

DSF exerts various biological effects by regulating multiple signalling pathways, and we mainly focused on the classical NLRP3/GSDMD pyroptosis pathway and the TLR4/NF-κB pathway. However, our research team is continuing to investigate other mechanisms and pathways through which DSF can treat ICH-induced tissue injury, such as by inhibiting oxidative stress and angiogenesis, to improve the therapeutic efficacy of DSF and to provide a better safety profile for its clinical application.

## Supplementary Information

Supplementary Figures.

## Data Availability

All data generated or analysed during this study are included in this published article.
