# Supplementary material for: Disulfiram attenuates cell and tissue damage and blood‒brain barrier dysfunction after intracranial haemorrhage by inhibiting the classical pyroptosis pathway
Source: Sci Rep. 2024 Sep 19;14:21860. doi: 10.1038/s41598-024-67118-2 (PMC11413208; doi:10.1038/s41598-024-67118-2)

Supplementary Figure S1

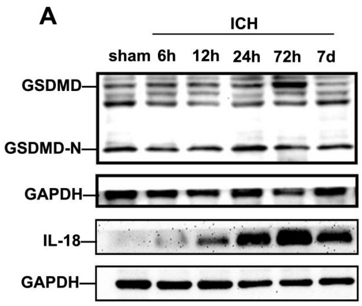

Figure 1A

The order of gel stripes in the blue box is sham, 6h- ICH,12h-ICH, 24h-ICH, 72h- ICH, 7d-ICH.

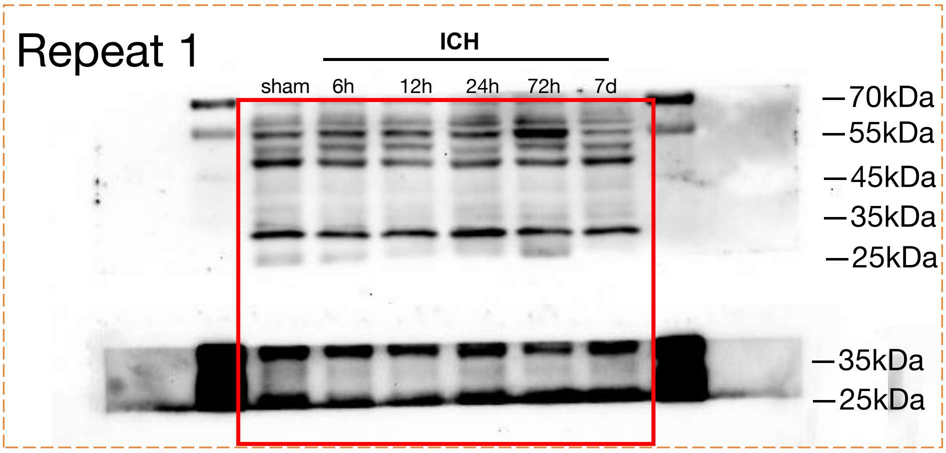

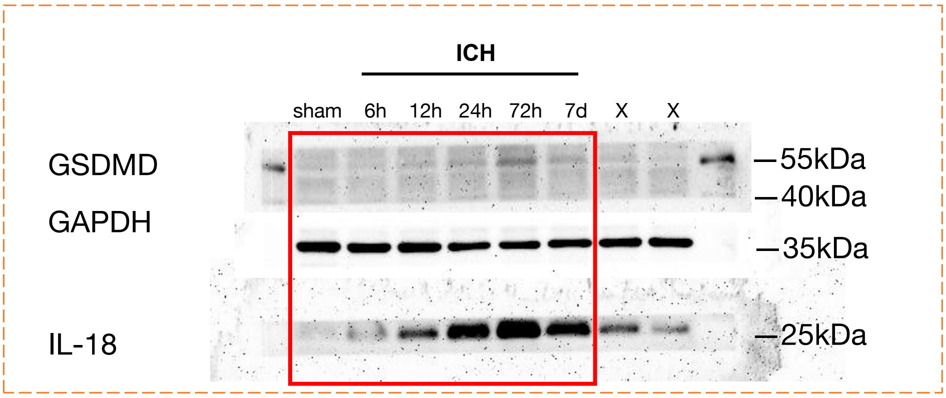

Repeat 2

Repeat 3

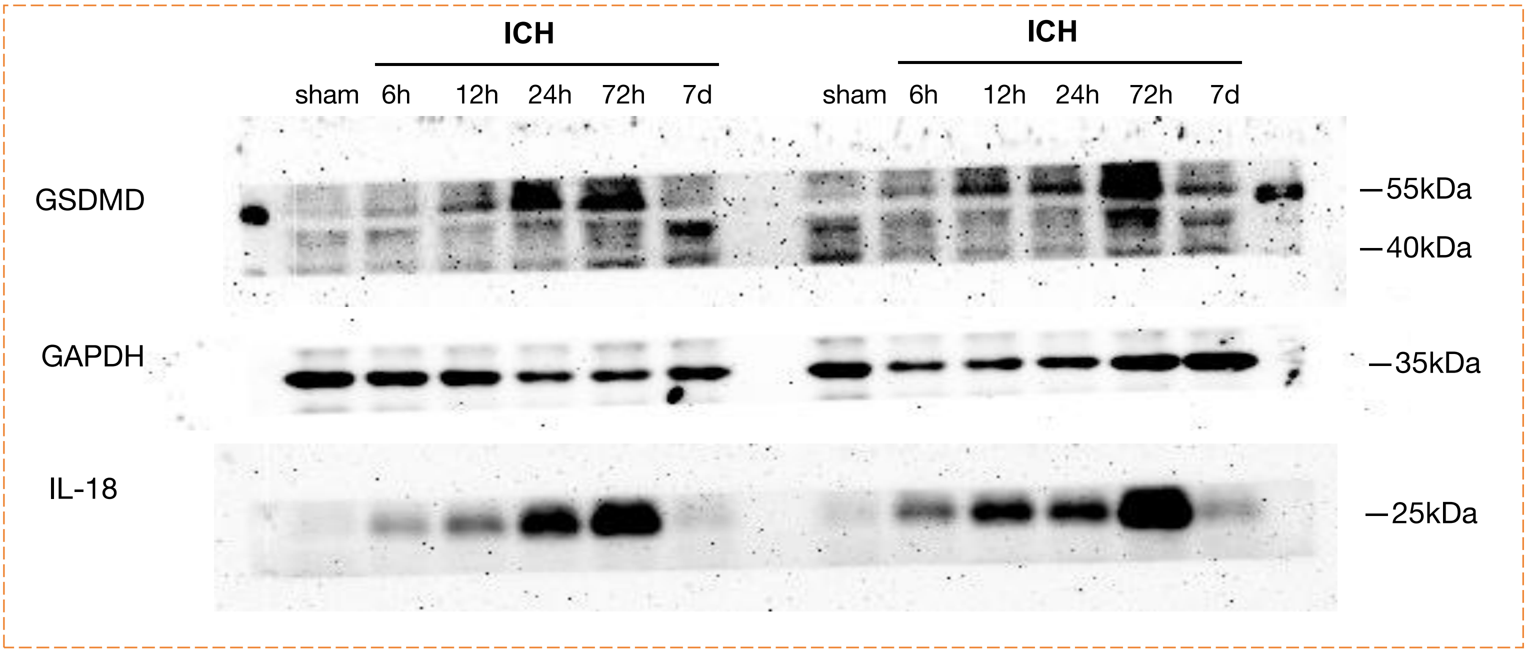

Supplementary Figure S1. Original Western blots of Figure 1A (red box circled). We completed the Western-blot experiment shown in this picture in the same gel. However, in order to incubate different primary antibodies, western blots were cropped prior to incubation with primary antibody hybridization. We repeated the experiment three times on a single gelatin plate.

Supplementary Figure S2

Repeat 2

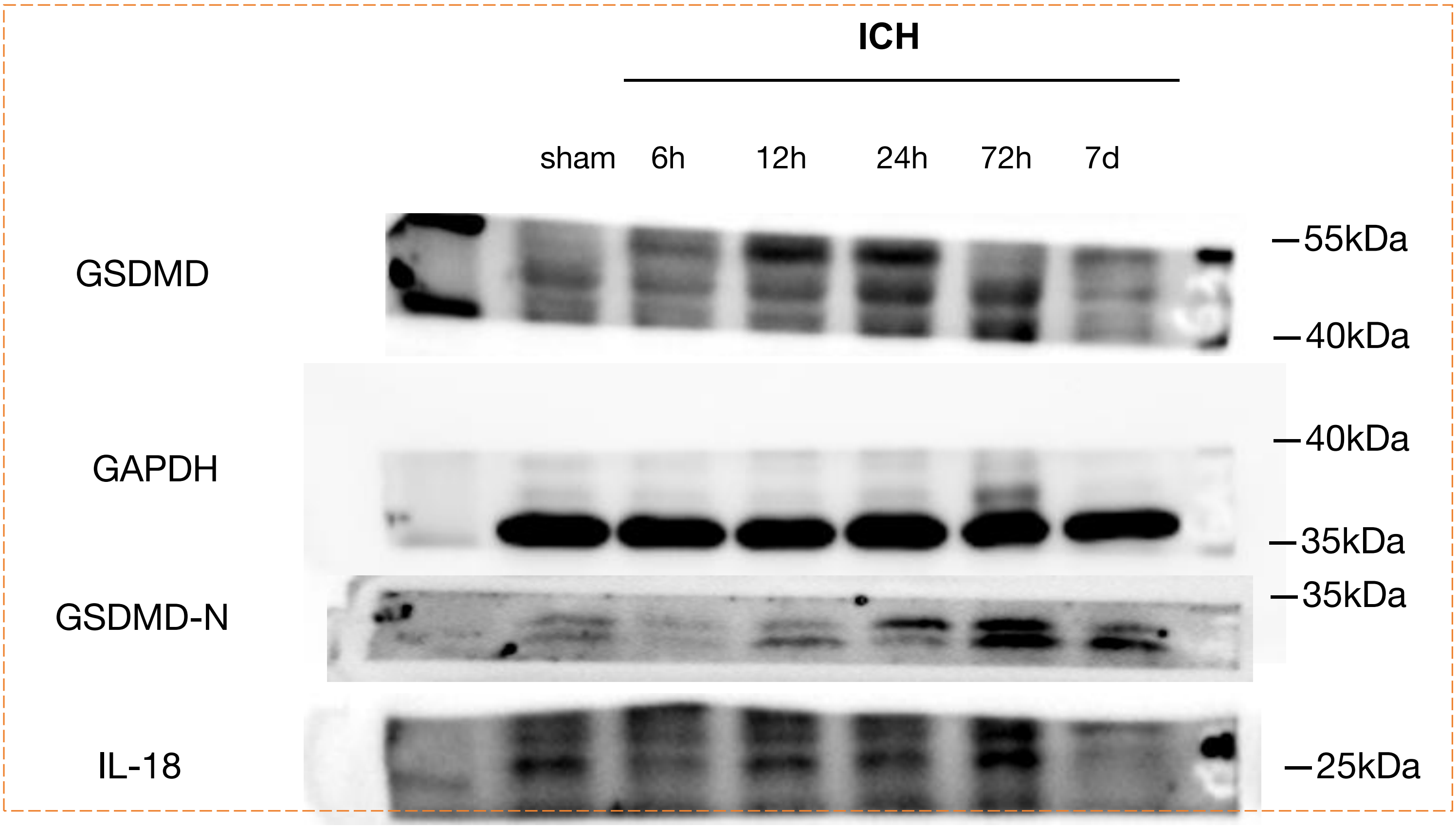

Repeat 3

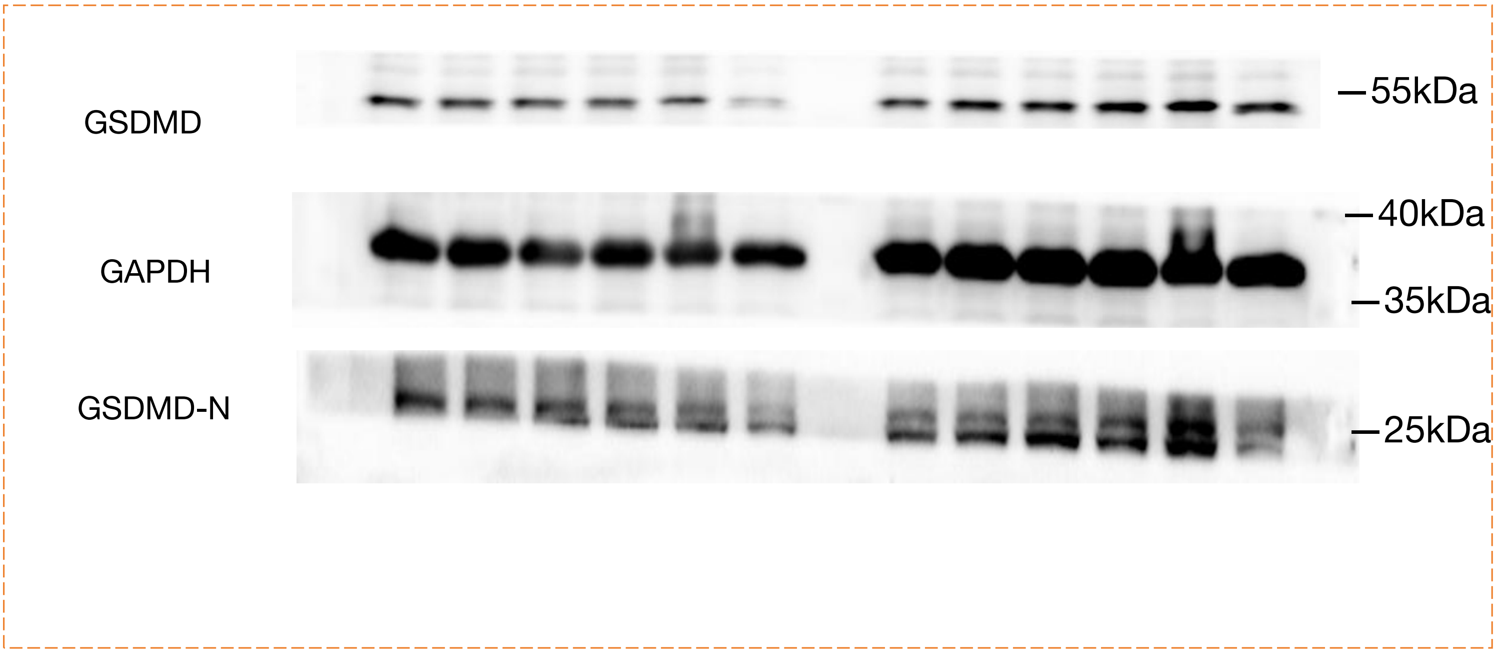

Supplementary Figure S2. Original Western blots of Figure 1A (red box circled). We completed the Western-blot experiment shown in this picture in the same gel. However, in order to incubate different primary antibodies, western blots were cropped prior to incubation with primary antibody hybridization. We repeated the experiment three times on a single gelatin plate.

Supplementary Figure S3

Repeat 1 Repeat 2

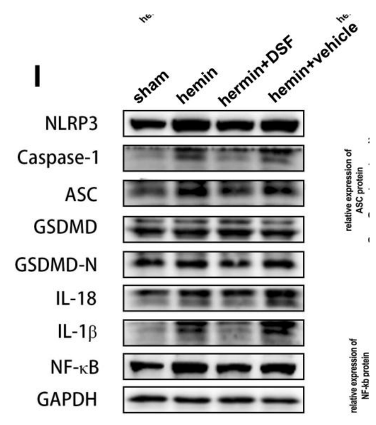

Figure 2I

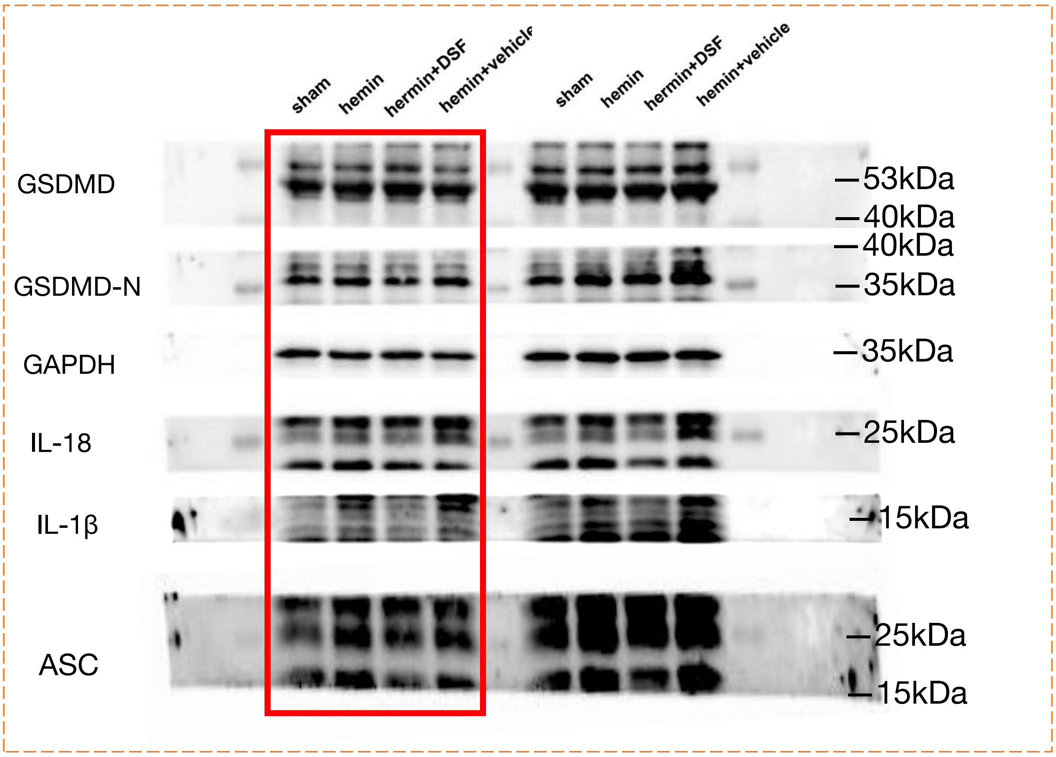

The order of gel stripes in the blue box is sham, hemin,hemin+DSF, hemin+vechicle.

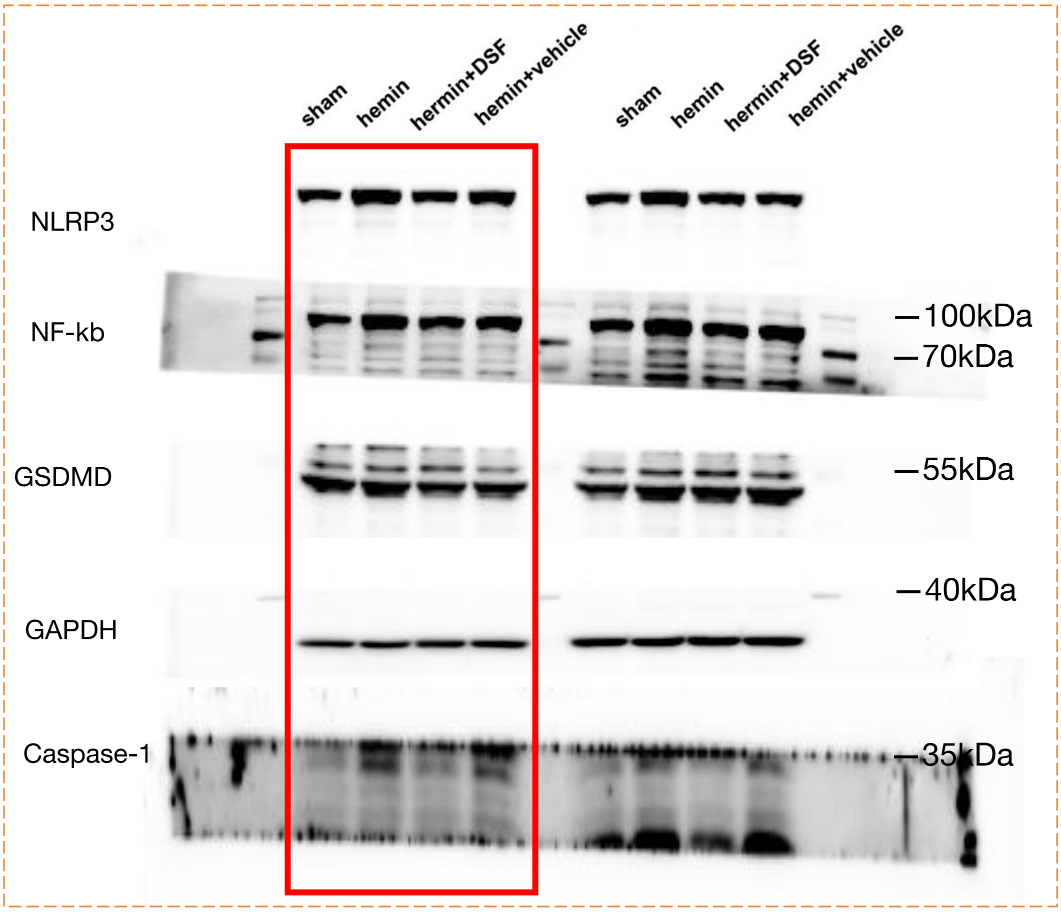

Supplementary Figure S3. Original Western blots of Figure 2I (red box circled). We completed the Western-blot experiment shown in this picture in the same gel. However, in order to incubate different primary antibodies, western blots were cropped prior to incubation with primary antibody hybridization. We repeated the experiment three times on a single gelatin plate.

Supplementary Figure S4

Repeat 3

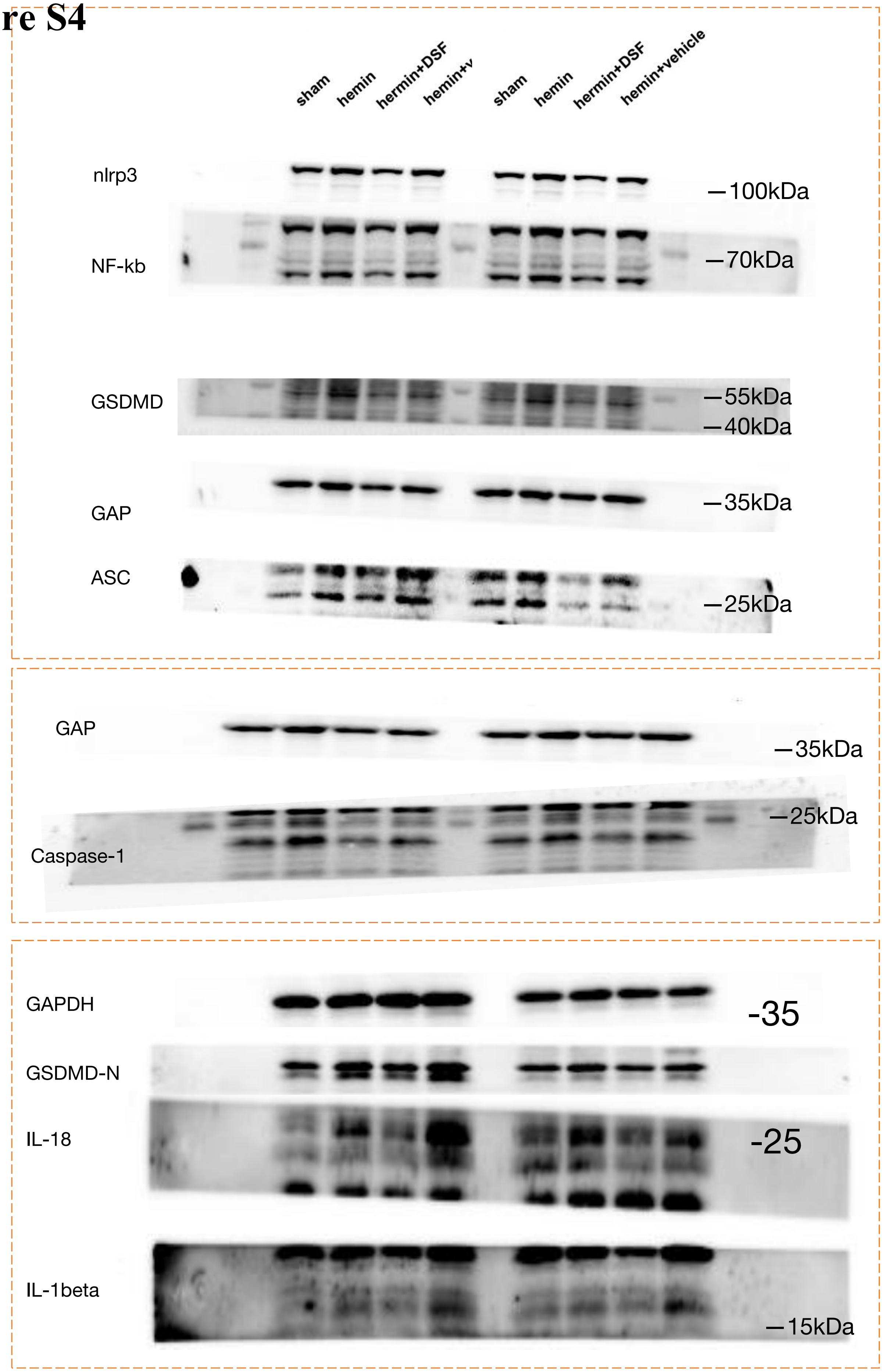

The order of gel stripes in the blue box is sham, hemin,hemin+DSF, hemin+vechicle.

Supplementary Figure S4. Original Western blots of Figure 2I . We completed the Western-blot experiment shown in this picture in the same gel. However, in order to incubate different primary antibodies, western blots were cropped prior to incubation with primary antibody hybridization. We repeated the experiment three times on a single gelatin plate.

Supplementary Figure S5

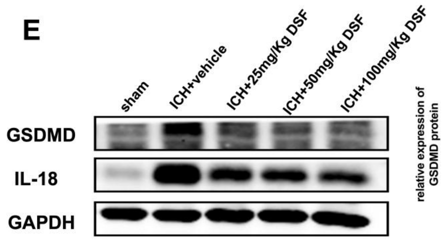

Figure 3E

The samples appear spaced because they were uploaded incorrectly, and the incorrect samples have been denoted with an X.

Repeat 1 Repeat 2

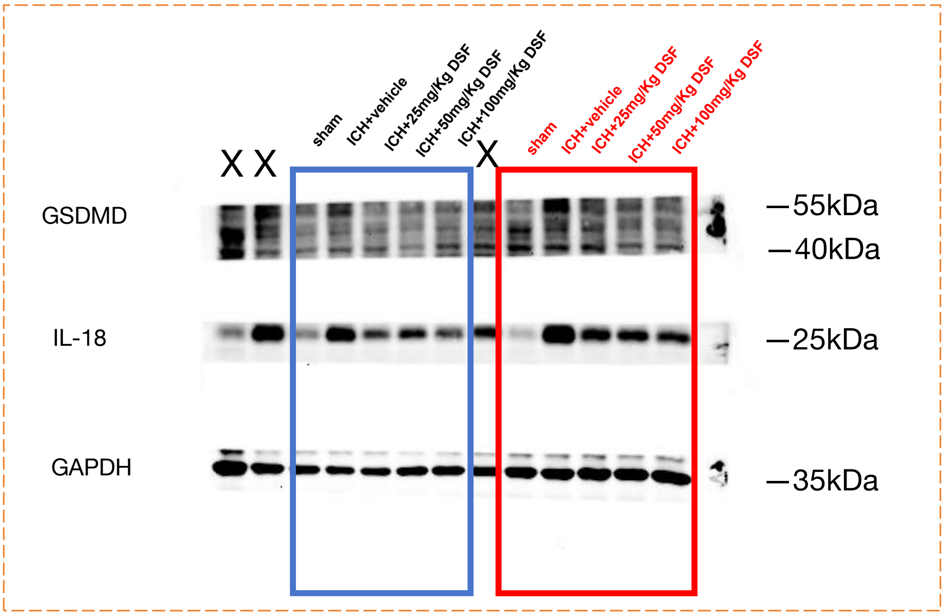

Repeat 3

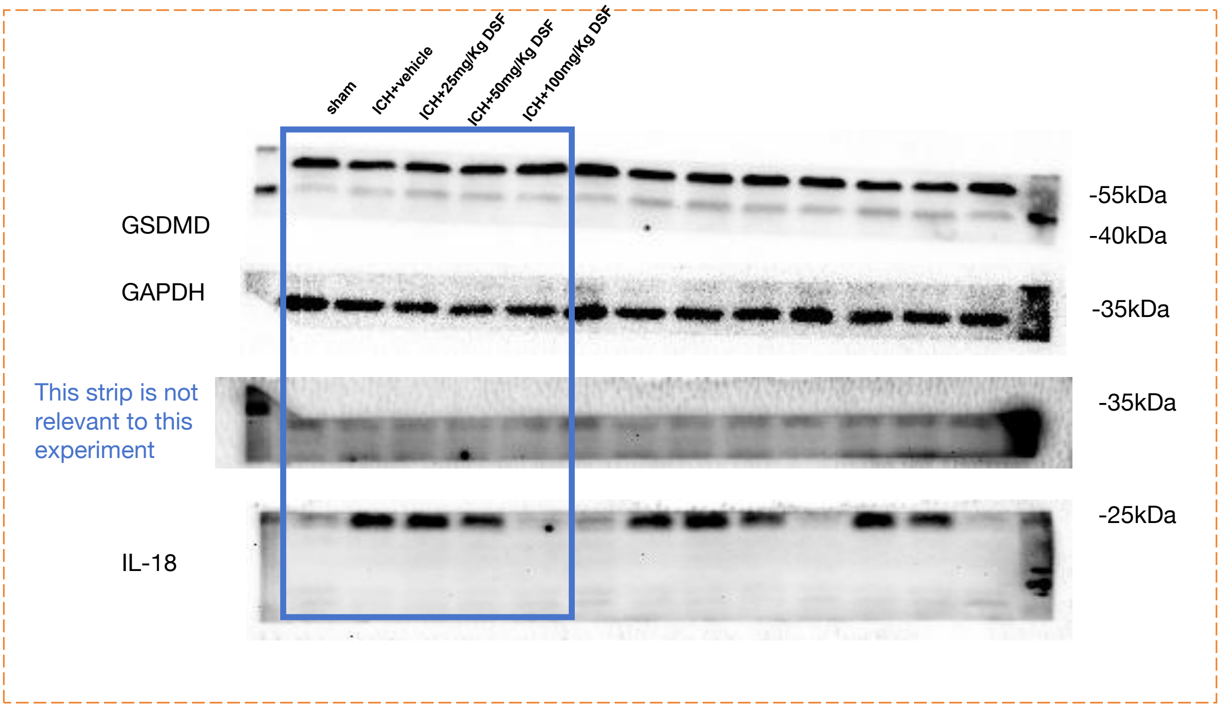

The order of gel stripes in the blue box is sham, ICH+vechicle,ICH+25mg/Kg DSF, ICH+50mg/KgDSF and ICH+100mg/KgDSF.

Supplementary Figure S5. Original Western blots of Figure 3E (red box circled). We completed the Western-blot experiment shown in this picture in the same gel. However, in order to incubate different primary antibodies, western blots were cropped prior to incubation with primary antibody hybridization.. We repeated the experiment three times on a single gelatin plate.

Supplementary Figure S6

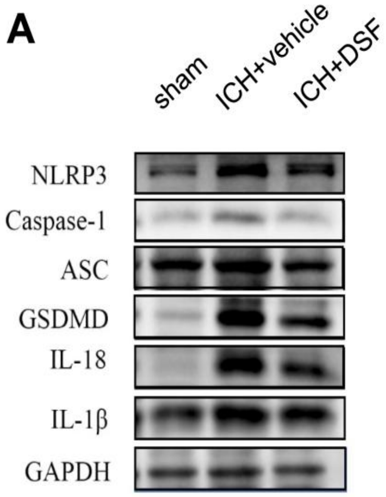

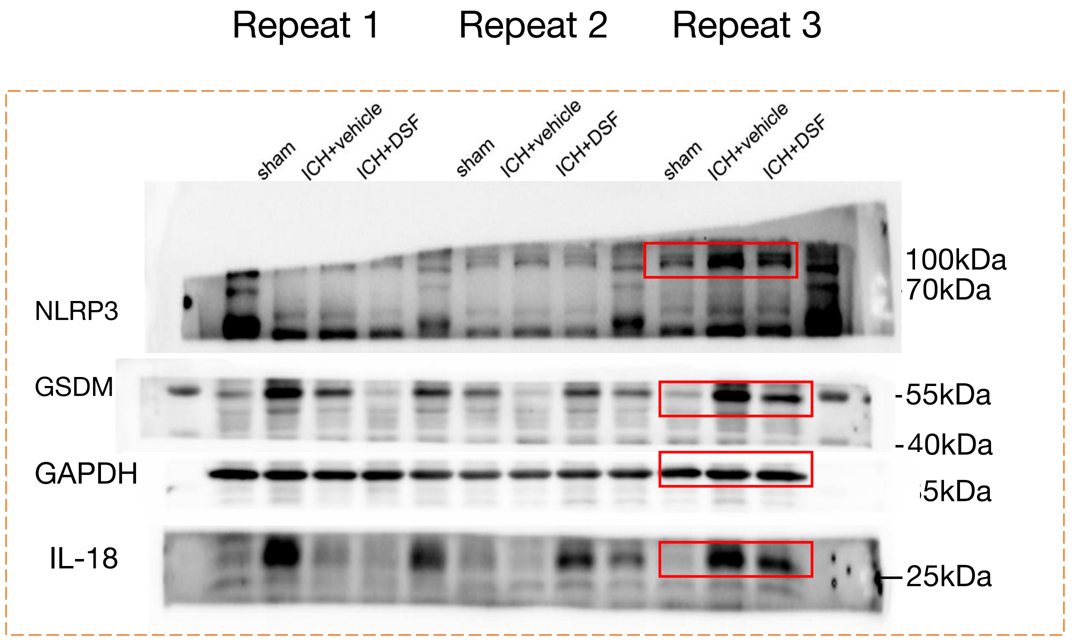

Figure 5A

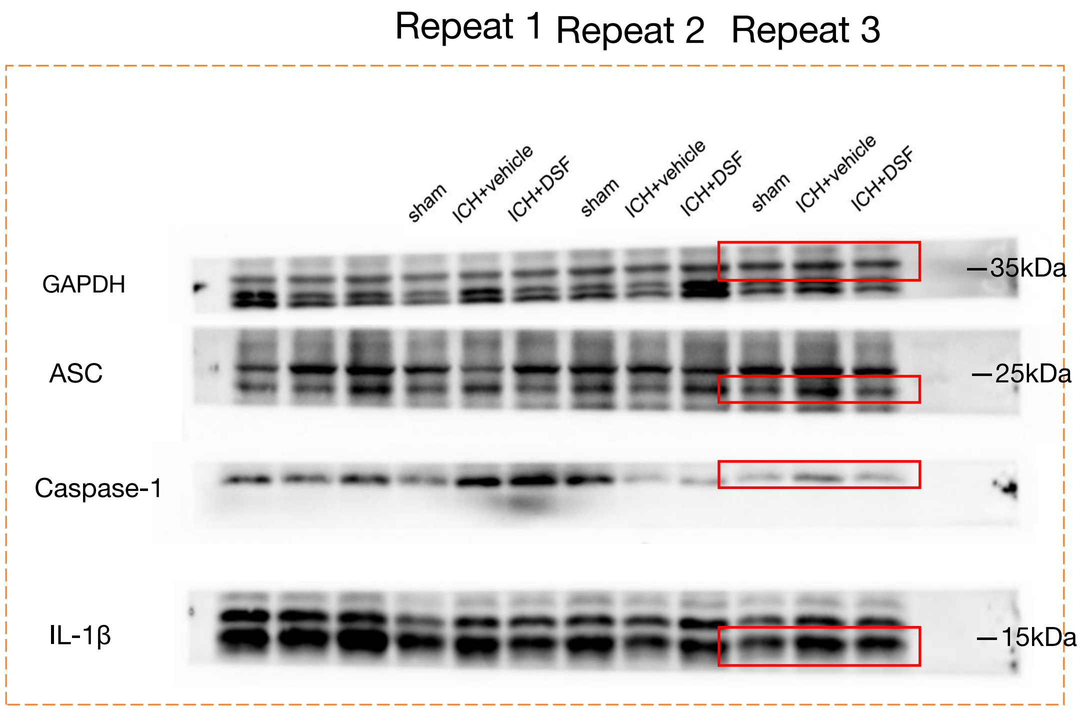

The order of gel stripes in the blue box is sham, ICH+vechicle and ICH+DSF.

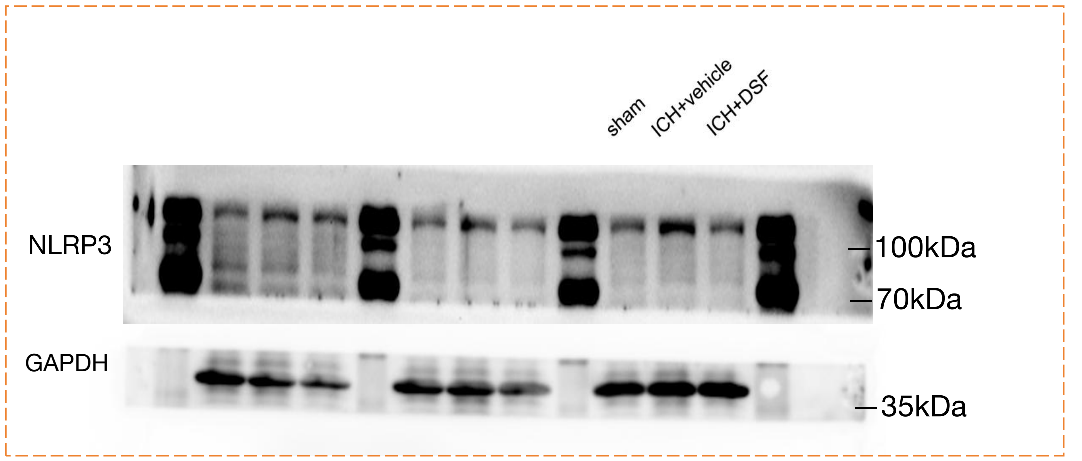

Supplementary Figure S6. Original Western blots of Figure 5A (red box circled). We completed the Western-blot experiment shown in this picture in the same gel. However, in order to incubate different primary antibodies, western blots were cropped prior to incubation with primary antibody hybridization.. We repeated the experiment three times on a single gelatin plate.

Supplementary Figure S7

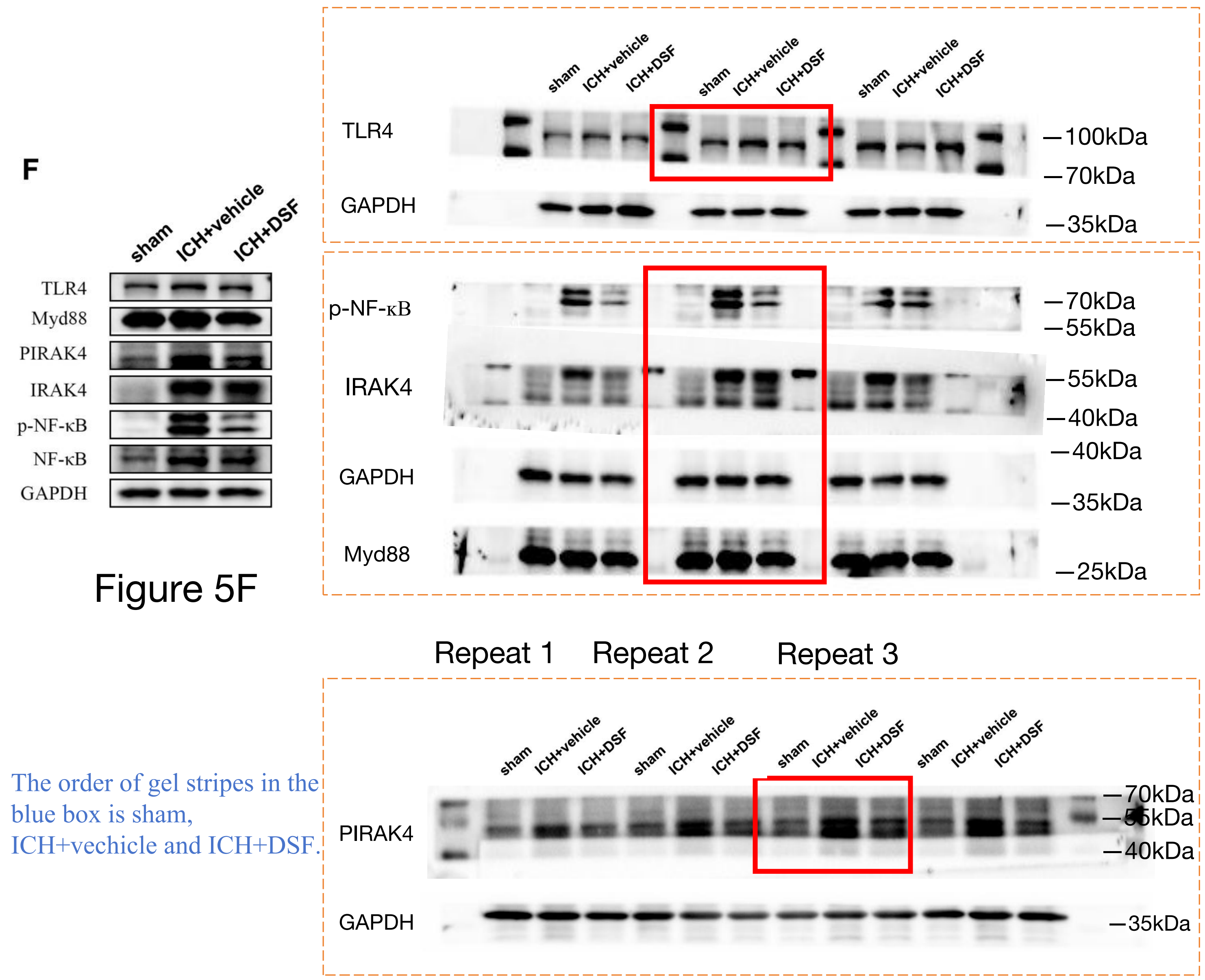

Supplementary Figure S7. Original Western blots of Figure 5F (red box circled). We completed the Western-blot experiment shown in this picture in the same gel. However, in order to incubate different primary antibodies, western blots were cropped prior to incubation with primary antibody hybridization.. We repeated the experiment three times on a single gelatin plate.

Supplementary Figure S8

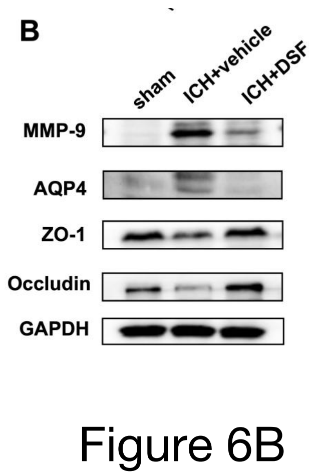

The order of gel stripes in the blue box is sham, ICH+vechicle and ICH+DSF.

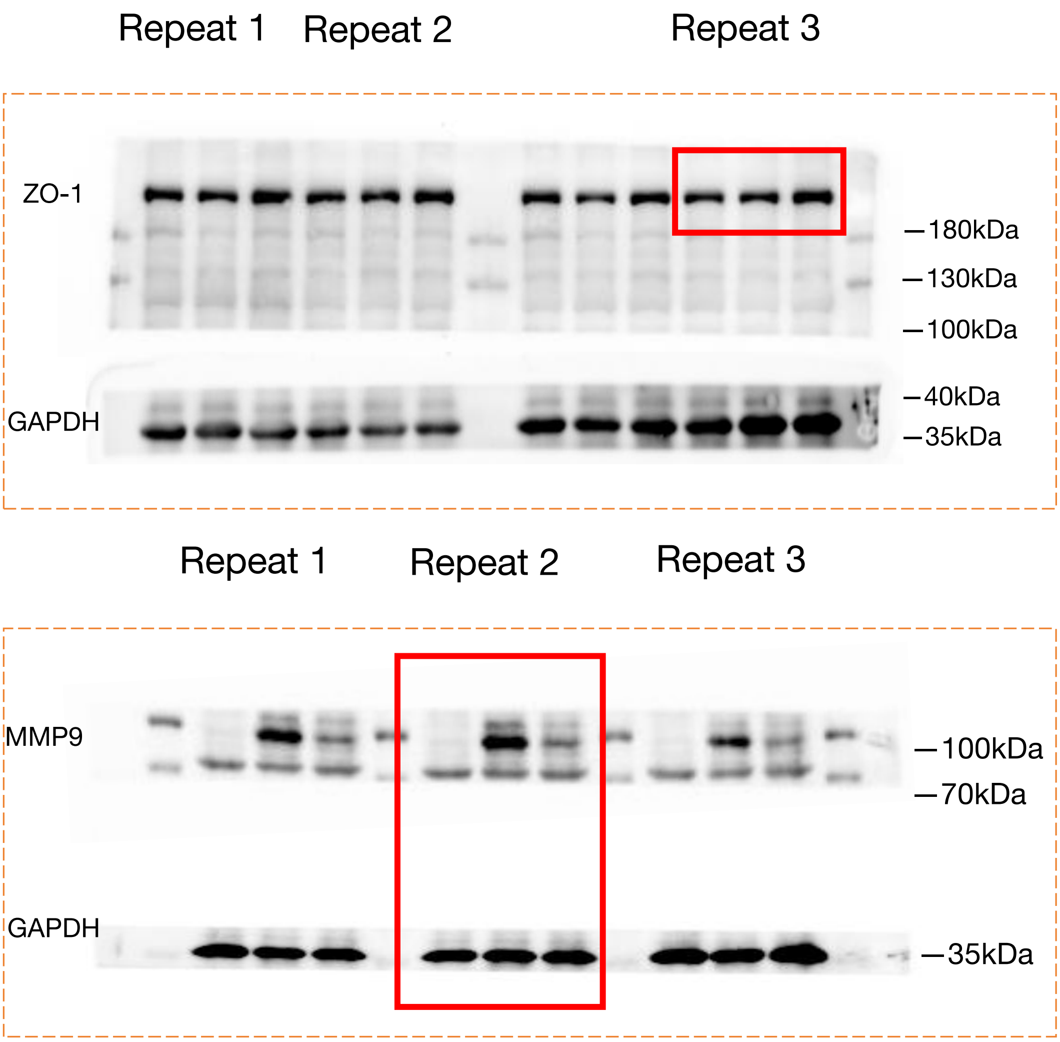

Supplementary Figure S8. Original Western blots of Figure 1A (red box circled). We completed the Western-blot experiment shown in this picture in the same gel. However, in order to incubate different primary antibodies, western blots were cropped prior to incubation with primary antibody hybridization.. We repeated the experiment three times on a single gelatin plate.

Supplementary Figure S9

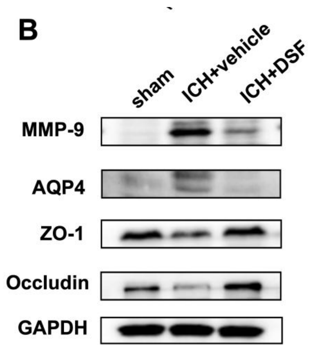

Figure 6B

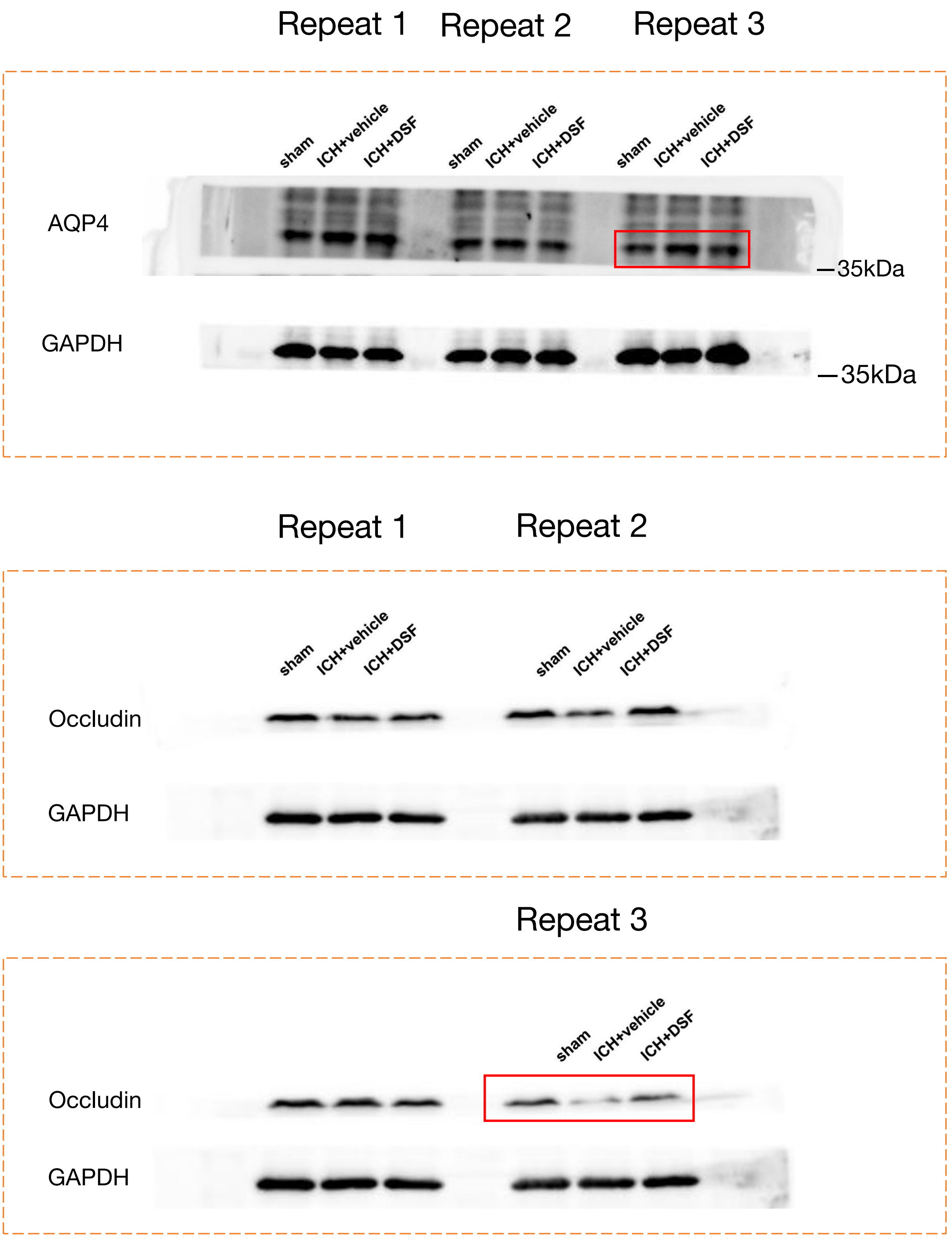

The order of gel stripes in the blue box is sham, ICH+vechicle and ICH+DSF.

Supplementary Figure S9. Original Western blots of Figure 6B (red box circled). We completed the Western-blot experiment shown in this picture in the same gel. However, in order to incubate different primary antibodies, western blots were cropped prior to incubation with primary antibody hybridization.. We repeated the experiment three times on a single gelatin plate.

Figure S1,2

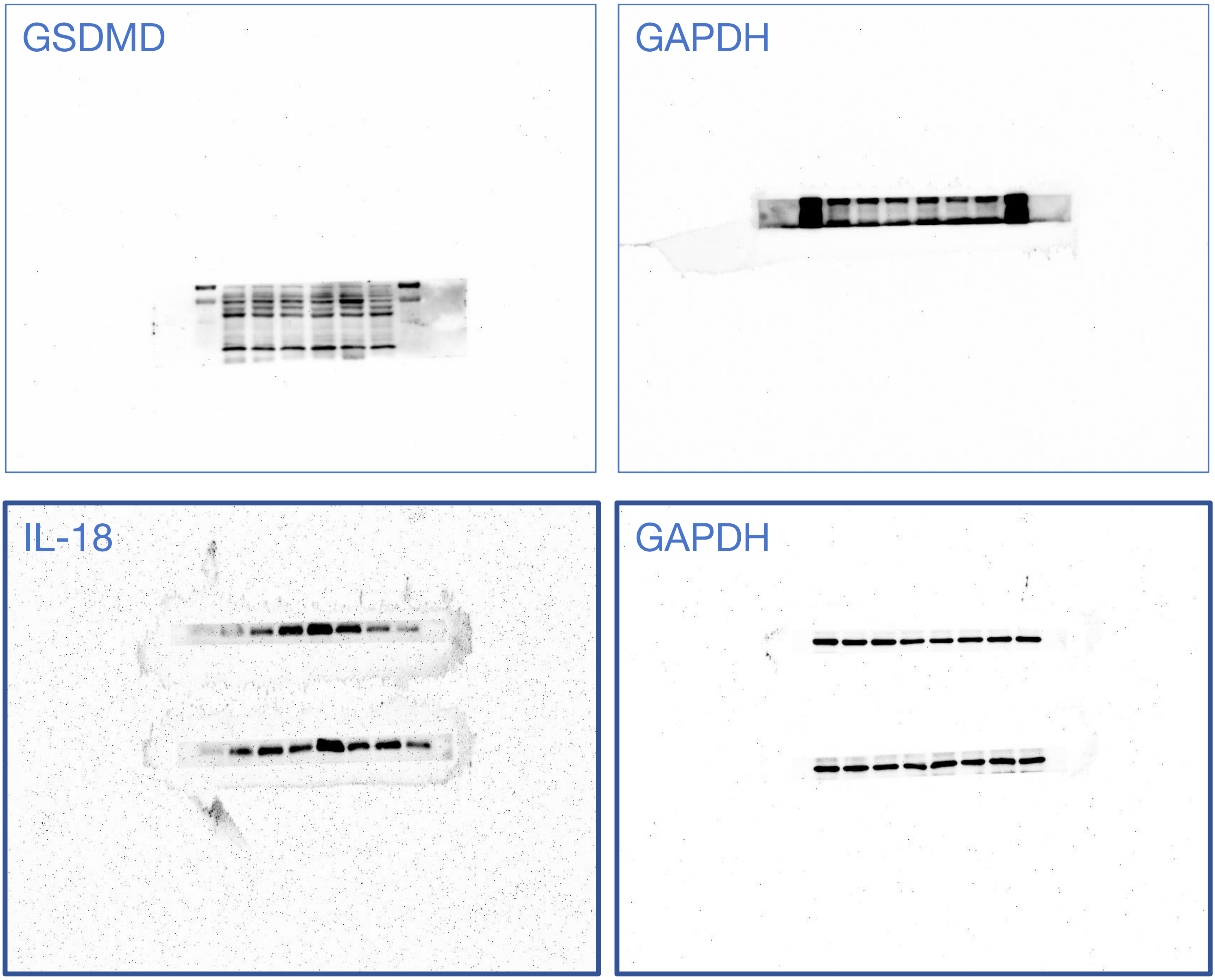

Figure S3,4

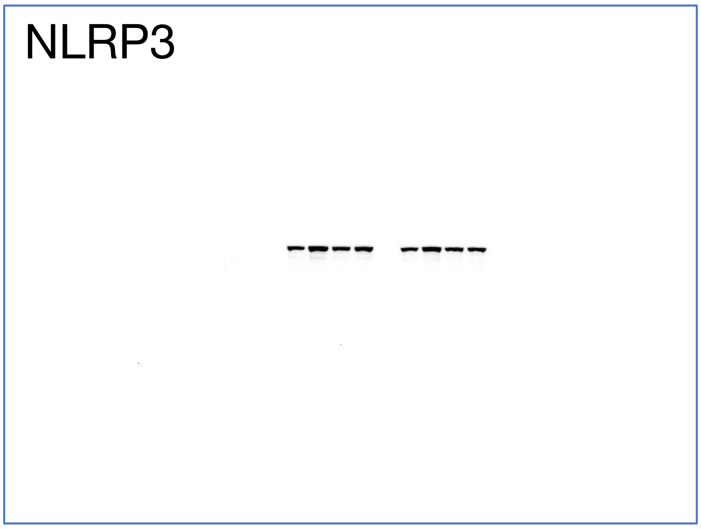

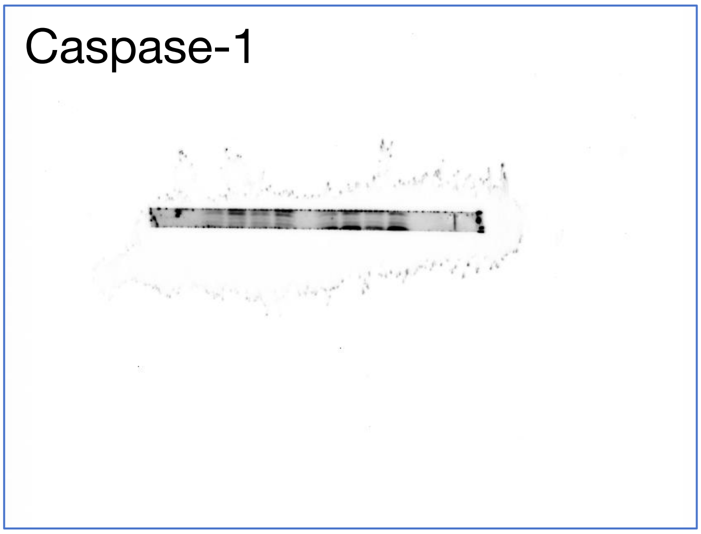

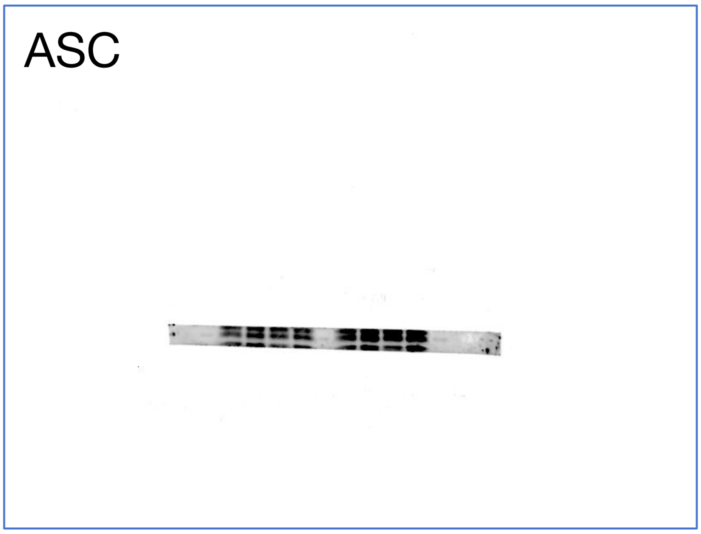

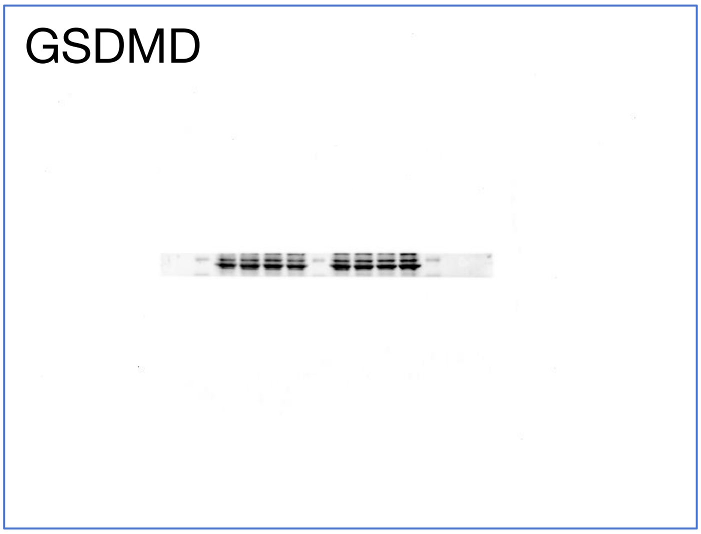

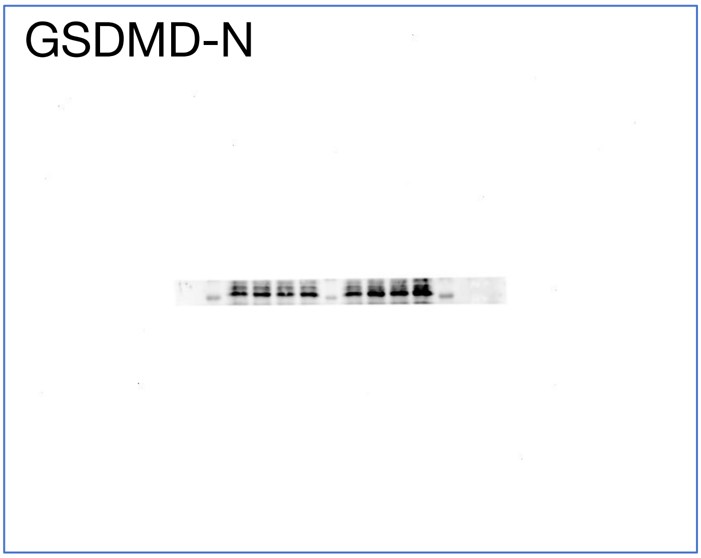

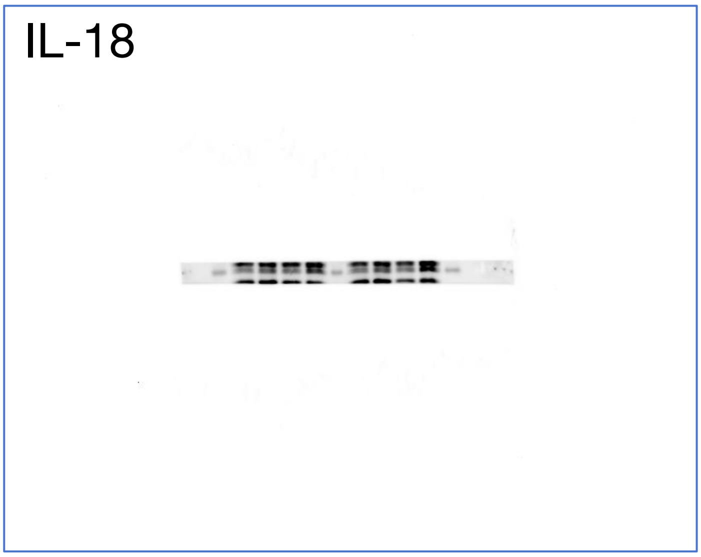

Figure S 3,4

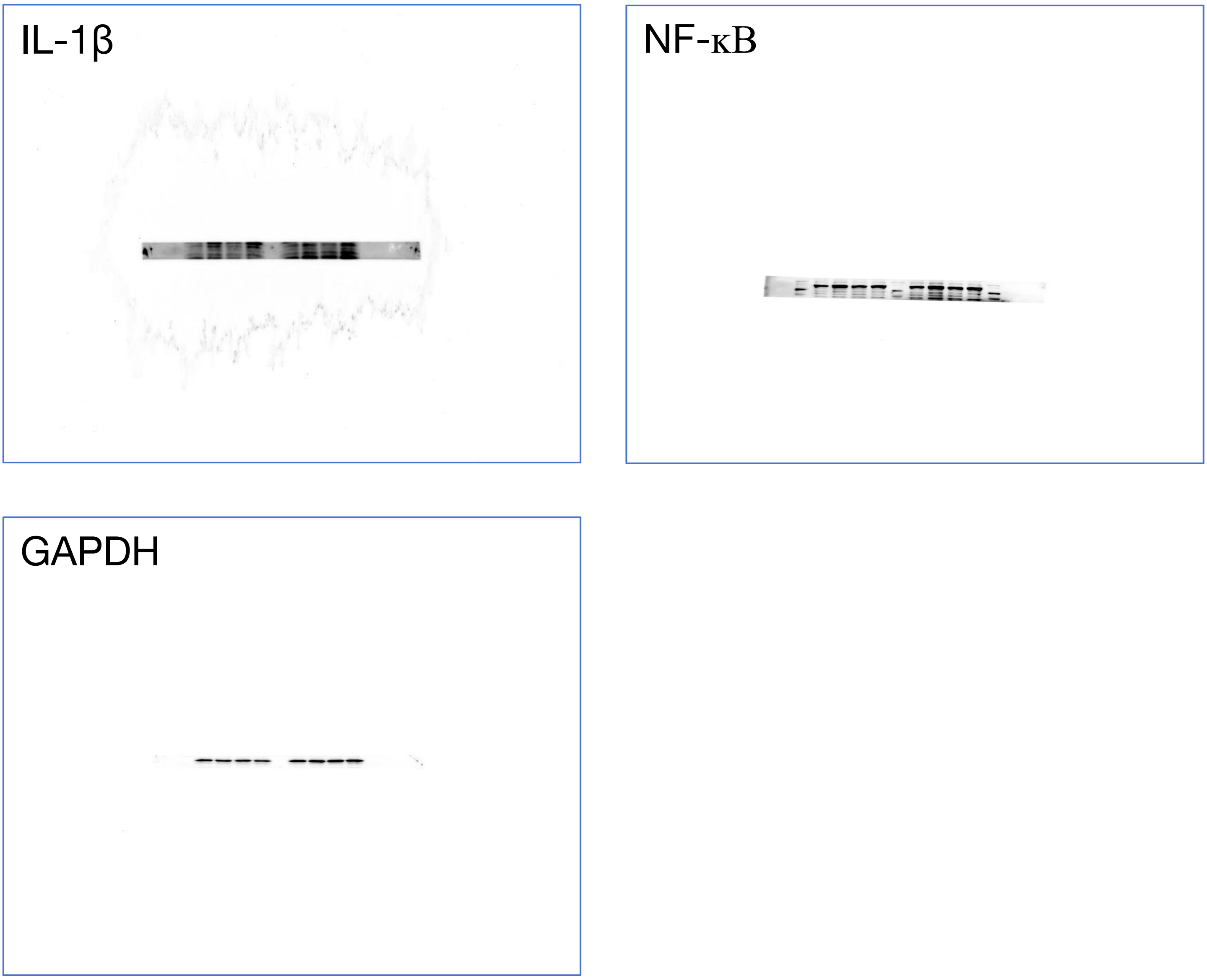

Figure S5

GSDMD

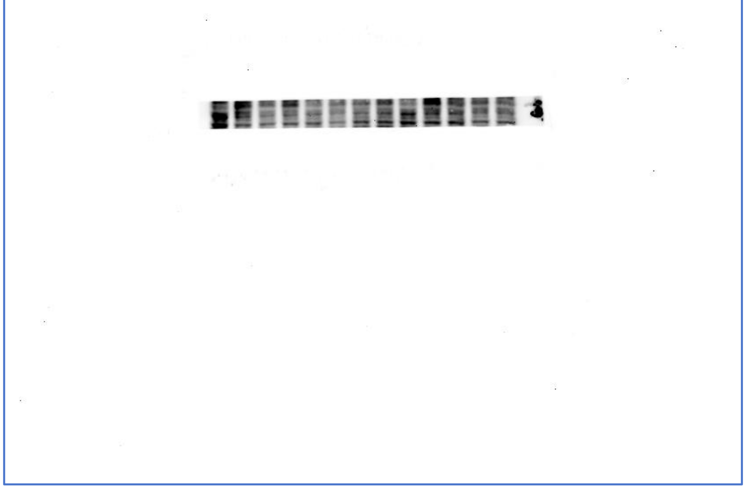

IL-18

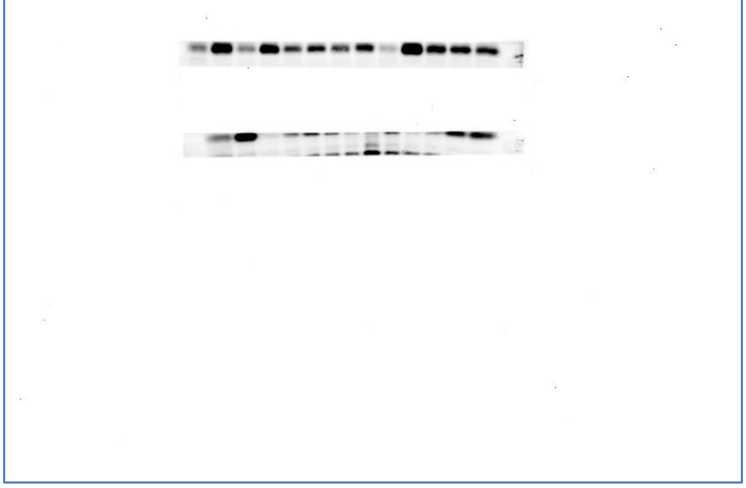

GAPDH

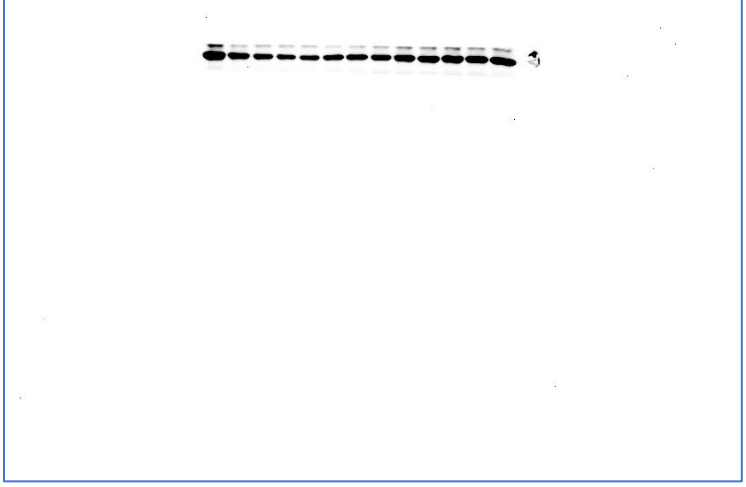

Figure S6

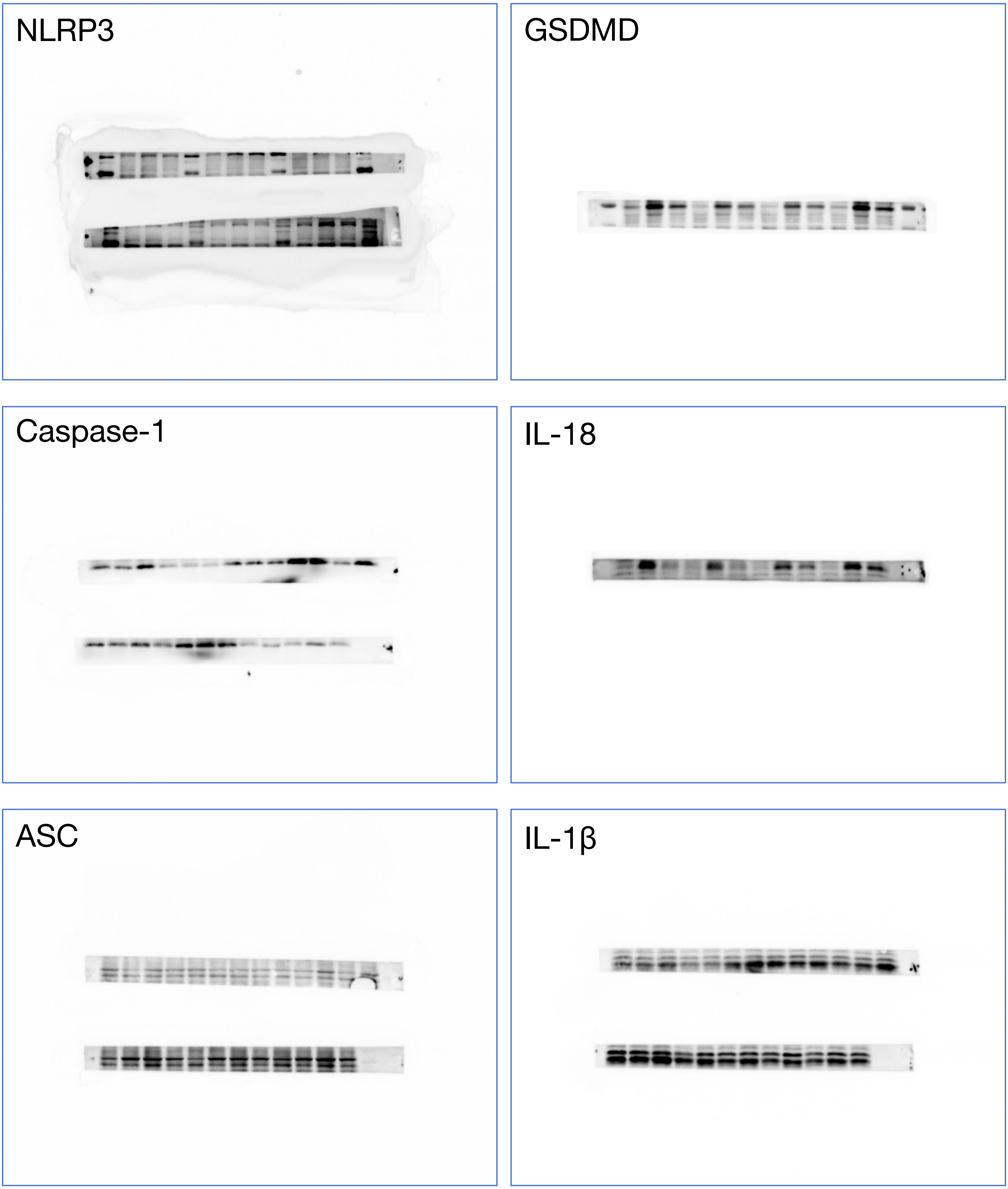

Figure S6

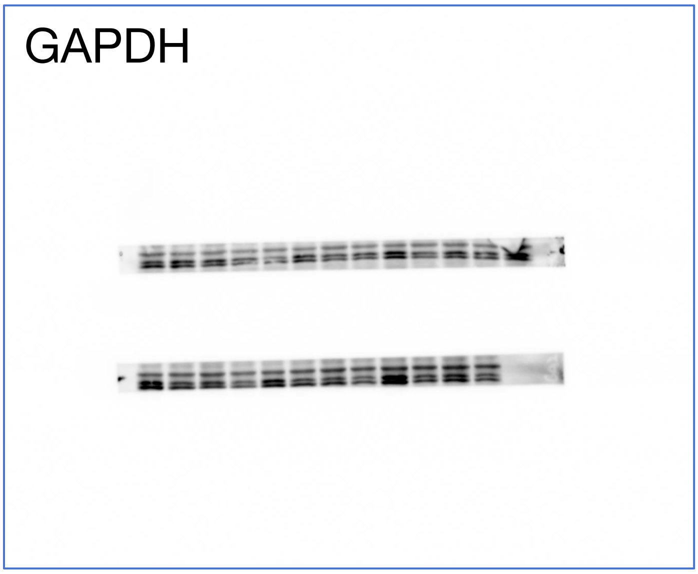

Figure S7

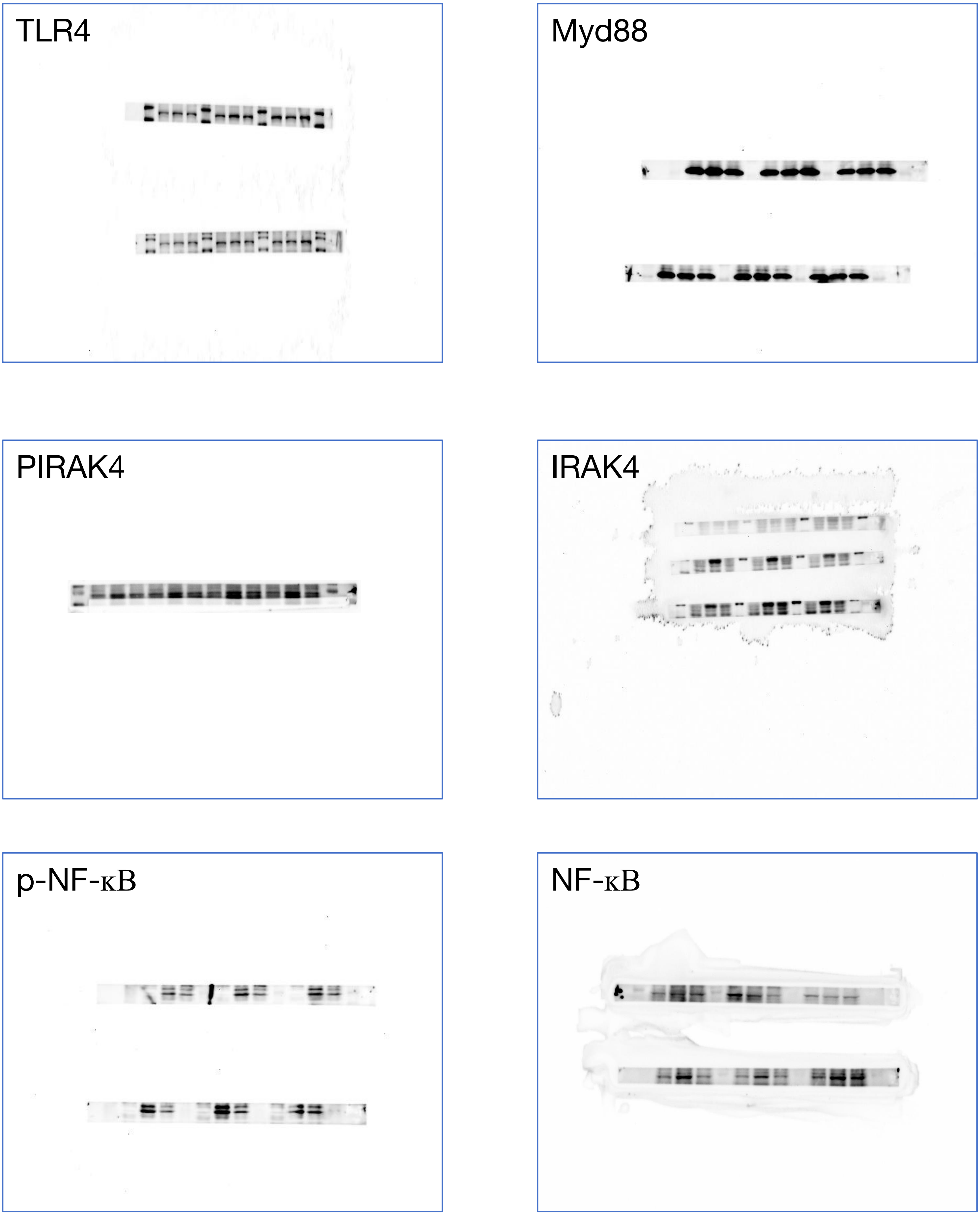

Figure S7

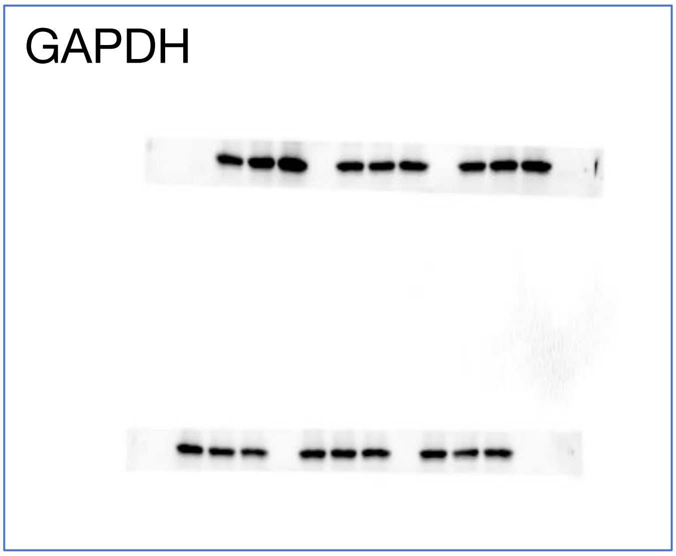

Figure S8,9

MMP9

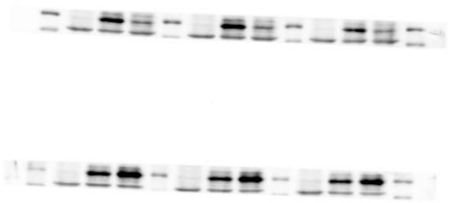

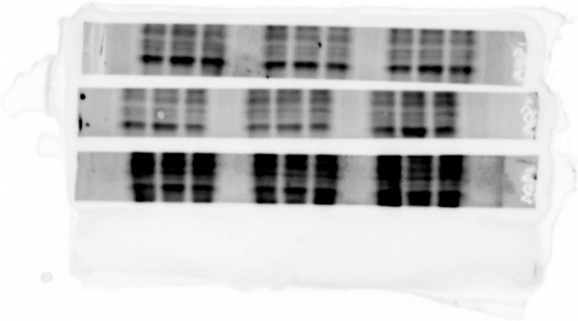

ZO-1

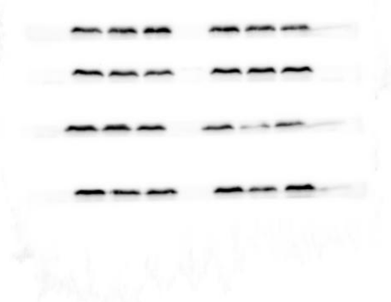

Occludin

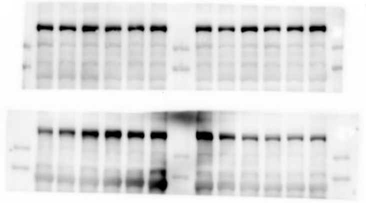

GAPDH

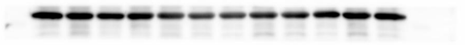

Supplement: Supplementary file 1 — Supplementary Figures. [file 41598_2024_67118_MOESM1_ESM.pdf]
